# A de novo dominant-negative variant is associated with OTULIN-related autoinflammatory syndrome

**DOI:** 10.1084/jem.20231941

**Published:** 2024-04-23

**Authors:** Yukiko Takeda, Masahiro Ueki, Junpei Matsuhiro, Erik Walinda, Takayuki Tanaka, Masafumi Yamada, Hiroaki Fujita, Shunichiro Takezaki, Ichiro Kobayashi, Sakura Tamaki, Sanae Nagata, Noriko Miyake, Naomichi Matsumoto, Mitsujiro Osawa, Takahiro Yasumi, Toshio Heike, Fumiaki Ohtake, Megumu K. Saito, Junya Toguchida, Junko Takita, Tadashi Ariga, Kazuhiro Iwai

**Affiliations:** 1 Department of Molecular and Cellular Physiology, Graduate School of Medicine, Kyoto University, Kyoto, Japan; 2 Department of Pediatrics, Faculty of Medicine and Graduate School of Medicine, Hokkaido University, Sapporo, Japan; 3 Department of Pediatrics, Graduate School of Medicine, Kyoto University, Kyoto, Japan; 4 Department of Food and Human Wellness, Rakuno Gakuen University, Ebetsu, Japan; 5 Department of Regeneration Science and Engineering, Institute for Life and Medical Sciences, Kyoto University, Kyoto, Japan; 6 Department of Fundamental Cell Technology, Center for iPS Cell Research and Application, Kyoto University, Kyoto, Japan; 7 Department of Human Genetics, Yokohama City University Graduate School of Medicine, Yokohama, Japan; 8 Department of Human Genetics, Research Institute, National Center for Global Health and Medicine, Tokyo, Japan; 9 Department of Clinical Application, Center for iPS Cell Research and Application, Kyoto University, Kyoto, Japan; 10 Institute for Advanced Life Sciences, Hoshi University, Tokyo, Japan

## Abstract

OTULIN-related autoinflammatory syndrome (ORAS), a severe autoinflammatory disease, is caused by biallelic pathogenic variants of OTULIN, a linear ubiquitin-specific deubiquitinating enzyme. Loss of OTULIN attenuates linear ubiquitination by inhibiting the linear ubiquitin chain assembly complex (LUBAC). Here, we report a patient who harbors two rare heterozygous variants of *OTULIN* (p.P152L and p.R306Q). We demonstrated accumulation of linear ubiquitin chains upon TNF stimulation and augmented TNF-induced cell death in mesenchymal stem cells differentiated from patient-derived iPS cells, which confirms that the patient has ORAS. However, although the de novo p.R306Q variant exhibits attenuated deubiquitination activity without reducing the amount of OTULIN, the deubiquitination activity of the p.P152L variant inherited from the mother was equivalent to that of the wild-type. Patient-derived MSCs in which the p.P152L variant was replaced with wild-type also exhibited augmented TNF-induced cell death and accumulation of linear chains. The finding that ORAS can be caused by a dominant-negative p.R306Q variant of OTULIN furthers our understanding of disease pathogenesis.

## Teaser

This study describes an OTULIN-related autoinflammatory syndrome (ORAS) patient with two rare heterozygous variants of *OTULIN* (p.P152L and p.R306Q); the latter is a de novo variant that acts in a dominant-negative manner to cause ORAS.

## Graphical Abstract

**Figure:**
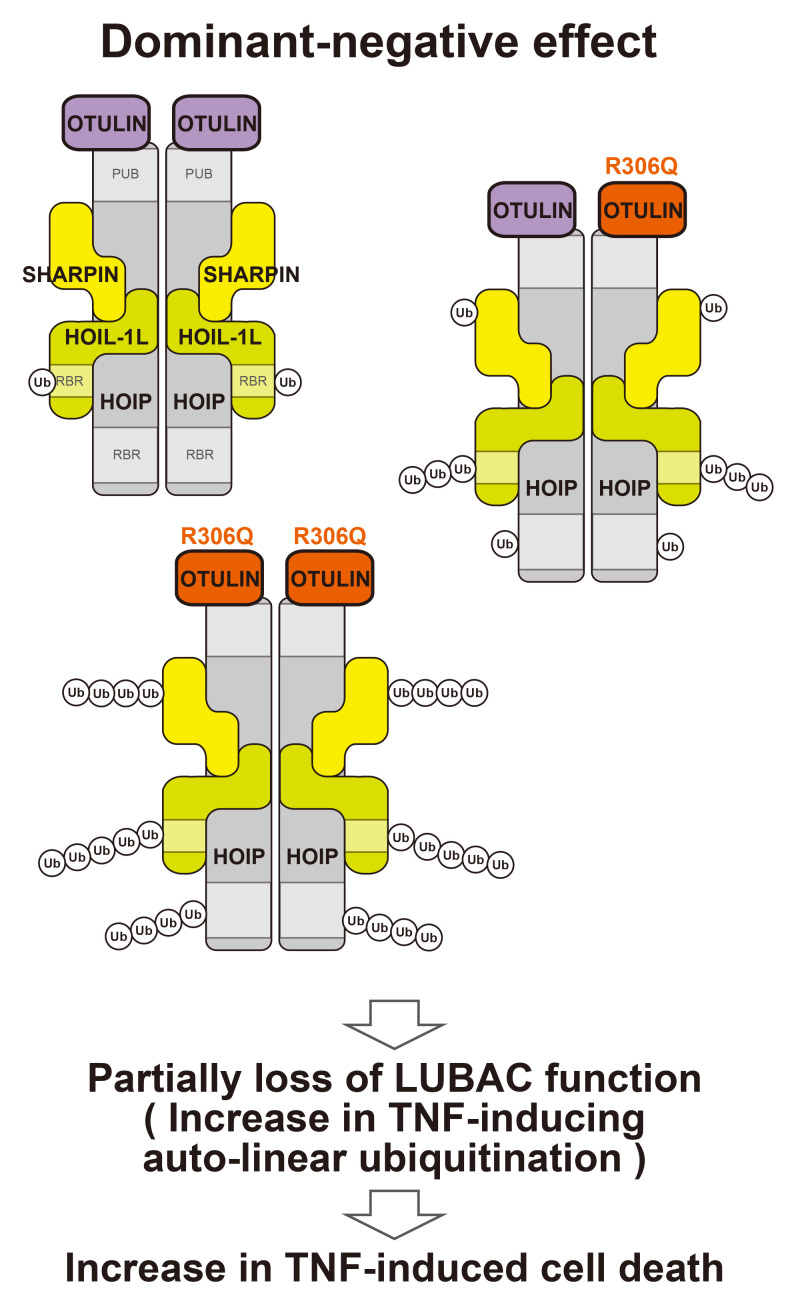

## Introduction

Timely activation and inactivation of innate immune responses are essential for tissue homeostasis and host defense against infection; dysregulation of these responses leads to autoinflammation (Aksentijevich and McDermott, 2017; Manthiram et al., 2017; Nigrovic et al., 2020). Autoinflammatory diseases are a heterogeneous group characterized by spontaneous onset of chronic inflammation despite the absence of identifiable proinflammatory factors (e.g., infection or tissue damage) (Aksentijevich and McDermott, 2017; Manthiram et al., 2017). More than 40 monogenic autoinflammatory diseases have been identified, and most affected genes are involved in the regulation of inflammatory cytokine signaling (Nigrovic et al., 2020).

The ubiquitin conjugation system is now recognized as an important reversible posttranslational modification system; the process involves the modification of proteins by the addition of ubiquitin chains (Dikic and Schulman, 2023; Hershko and Ciechanover, 1998). Cells possess a variety of ubiquitin chains, which have been thought to be linked via one of the seven lysine residues within ubiquitin (Hershko and Ciechanover, 1998). Previously, we identified a new type of ubiquitin chain (the linear ubiquitin chain) linked by an N-terminal methionine residue (Kirisako et al., 2006). So far, the linear ubiquitin chain assembly complex (LUBAC), which comprises catalytic HOIP and accessory subunits HOIL-1L and SHARPIN, is the only ubiquitin ligase that generates linear ubiquitin chains specifically (Kirisako et al., 2006; Tokunaga et al., 2011). LUBAC-mediated linear ubiquitination plays important functions in signal transduction, particularly within the TNF signaling pathway (Iwai et al., 2014; Tokunaga et al., 2009, 2011).

Abnormal or excessive TNF signaling triggers autoinflammation, which can be suppressed by TNF-blocking agents (Jarosz-Griffiths et al., 2019; Nigrovic et al., 2020). TNF triggers the assembly of the TNF receptor-1 (TNFR1) signaling complex, called Complex I, which includes RIPK1 and cIAP (Fuseya and Iwai, 2021; Peltzer and Walczak, 2019; van Loo and Bertrand, 2023). LUBAC is recruited to Complex I and activates IKK via linear ubiquitination of NEMO within the IKK complex, leading to activation of NF-κB (Fujita et al., 2014). Conversely, RIPK1 and other components of Complex I dissociate from TNFR1 to form a cytosolic complex called Complex II, which induces programmed cell death (apoptosis and necroptosis) (Fuseya and Iwai, 2021; Peltzer and Walczak, 2019; van Loo and Bertrand, 2023). Ubiquitination of RIPK1 suppresses the transition to Complex II (Kist et al., 2021; Zhang et al., 2019), and LUBAC prevents cell death by linearly ubiquitinating RIPK1 and other proteins within Complex I, thereby inhibiting the formation of Complex II (Fuseya et al., 2020; Tu et al., 2021).

Among the 90 deubiquitinating (DUB) enzymes in humans, OTULIN is the only one that cleaves linear ubiquitin chains specifically (Keusekotten et al., 2013; Rivkin et al., 2013). Since OTULIN interacts constitutively with LUBAC, OTULIN was first thought to suppress linear ubiquitin chain-mediated signaling by cleaving the linear ubiquitin chains generated by LUBAC (Elliott et al., 2014; Schaeffer et al., 2014; Takiuchi et al., 2014). However, we now know that OTULIN regulates LUBAC activity together with the HOIL-1L subunit (Fuseya et al., 2020; Heger et al., 2018). LUBAC function is attenuated by linear auto-ubiquitination, which is achieved by auto-mono-ubiquitination of LUBAC subunits by HOIL-1L, followed by conjugation of linear chains to the ubiquitin moieties on LUBAC subunits by the catalytic HOIP subunit, resulting in attenuated LUBAC activity (Fuseya et al., 2020). OTULIN counteracts HOIL-1L/HOIP-mediated suppression of LUBAC by digesting the linear ubiquitin chains on LUBAC (Fuseya et al., 2020; Heger et al., 2018). Therefore, attenuation of OTULIN function increases TNF-mediated cell death (Fuseya et al., 2020; Heger et al., 2018).

Pathogenic variants of genes encoding components of the linear ubiquitination system cause autoinflammatory diseases. Biallelic *HOIL-1L* (*RBCK1*) or *HOIP* (*RNF31*) variants result in autoinflammation with immunodeficiency (Aksentijevich and Zhou, 2017; Boisson et al., 2012, 2015). Moreover, homozygous or compound heterozygous *OTULIN* variants cause an autoinflammatory disease called otulipenia or OTULIN-related autoinflammatory syndrome (ORAS) in an autosomal recessive fashion (Damgaard et al., 2016, 2019; Nabavi et al., 2019; Zhou et al., 2016; Zinngrebe et al., 2022). ORAS is characterized by fever and autoinflammation, sterile neutrophilic dermatitis, and failure to thrive. Recent studies show that anti-TNF therapy is a viable treatment for ORAS (Damgaard et al., 2019; Zinngrebe et al., 2022). Indeed, fibroblasts from ORAS patients exhibit a subtle reduction in TNF-mediated NF-κB activation and increased sensitivity to TNF-induced cell death (Damgaard et al., 2019; Zinngrebe et al., 2022). Here, we report the case of a girl with symptoms consistent with ORAS, in whom the disease appears to be triggered by a heterozygous dominant-negative variant of *OTULIN*.

## Results

### A female patient presenting with ORAS-like symptoms

**Figure 1.**
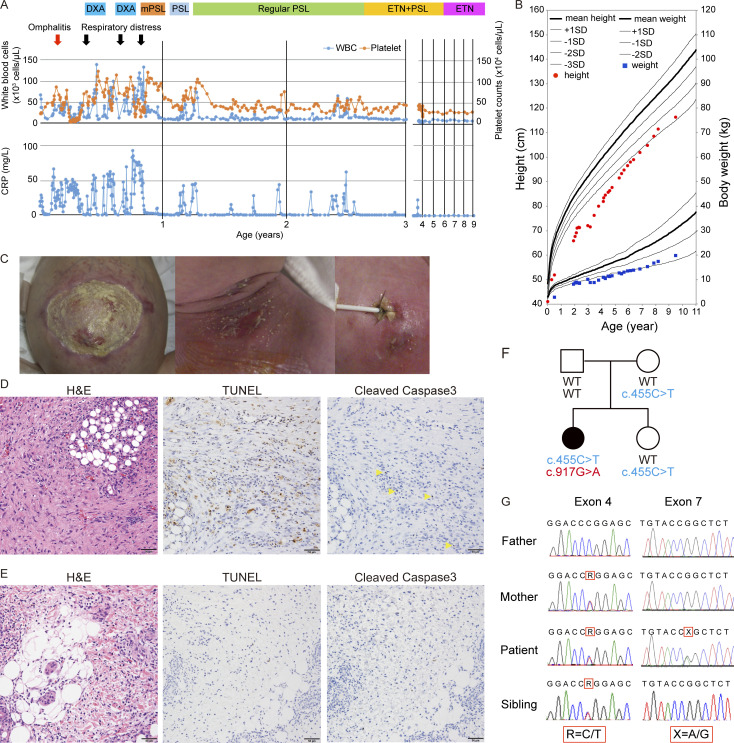
**Rare compound heterozygous variants of *OTULIN* identified in a girl with ORAS-like symptoms. (A)** Characteristic manifestations, treatments, and laboratory data (WBC counts, platelet counts, and CRP levels) from birth to the present day. DXA, dexamethasone; mPSL, methylprednisolone; PSL, prednisolone; ETN, etanercept. **(B)** Plot of height (cm) versus body weight (kg) of the patient from birth to the present day. **(C)** Images of omphalitis following surgical treatment (left); erythema nodosum and pustulosis around the site of a needle puncture for central venous catheter insertion (middle); and erythema nodosum with pus around the region of central venous catheter (right). **(D and E)** Pathological examination of omphalitis specimen after surgical debridement at the age of 1 mo (D), and erythema nodosum on the left upper arm at 1 year of age (E). Specimens were stained with H&E (left), TUNEL (middle), or an anti-cleaved caspase-3 antibody (right). Yellow arrowheads point to cleaved caspase-3–positive cells. Scale bars: 50 µm. **(F)** Pedigree and identified genotypes in the family, along with *OTULIN* variants. The patient (denoted by the solid symbol) with inflammatory symptoms harbors c.917G>A and c.455C>T substitutions in *OTULIN*. ○, female; □, male. The mother and the patient’s sibling also harbor the c.455C>T substitution; c.917G>A is a de novo variant. **(G)** Direct sequencing of *OTULIN* variants identified in the family.

A Japanese girl exhibited persistent fever with an increased white blood cell (WBC) count, elevated serum C-reactive protein (CRP) levels, skin manifestations (i.e., aseptic pustulosis and erythema nodosum), and physical developmental delay (Fig. 1, A and B; and Clinical description). She developed severe inflammatory symptoms shortly after birth; these symptoms responded partially to antibiotic treatment, although no pathogenic bacteria were detected in blood and spinal fluid cultures. At the age of 1 mo, she developed refractory omphalitis and pustulosis, possibly caused by methicillin-susceptible *Staphylococcus aureus* (*S*. *aureus*), and underwent surgical debridement because the antibiotic-refractory inflammation extended to most of her abdominal wall (Fig. 1 C, left panel). Histopathologic examination of the surgically removed specimen revealed massive infiltration by leukocytes (mainly neutrophils) and inflammatory granuloma formation (Fig. 1, D and E). In addition, significant cell death was confirmed by TdT-mediated dUTP-biotin nick end labeling (TUNEL) staining and cleaved caspase-3 staining (Fig. 1, D and E). She also experienced three episodes of acute respiratory distress syndrome (ARDS) after 3 mo of age. There was a significant increase in serum IL-1β and IL-6 concentrations during the first episodes of ARDS; however, high-dose prednisolone ameliorated the inflammatory symptoms, reduced cytokine levels (Table S1), and decreased cell death (as shown by TUNEL staining and cleaved caspase-3 staining; Fig. 1, D and E). However, fever and inflammation relapsed, and she developed novel skin nodules after reducing the dose of steroid (Fig. 1 A). She also exhibited severe physical developmental delay (Fig. 1 B). Also, at 1 year of age, diffuse infiltration of neutrophils and mononuclear cells was observed in the dermis in the area of erythema nodosum in her left upper arm (Fig. 1 E). We then suspected that she was suffering from inborn errors of immunity (IEIs). Whole-exome sequencing (WES) of samples from the patient and her parents identified two heterozygous variants of the *OTULIN* gene (NM_138348.6: c.455C>T and c.917G>A) (Table S2 and Fig. 1, F and G). Both base substitutions were accompanied by amino acid substitutions: c.455C>T p.P152L and c.917G>A p.R306Q (Tables 1 and S2). Complementary DNA (cDNA) cloning studies confirmed that these variants in the patient were in different alleles. p.P152L (inherited from the mother) is a relatively rare variant in gnomAD (51 of 1,611,754 alleles, no homozygous pattern) (Karczewski et al., 2020), Bravo (3 of 125,568 alleles, no homozygous pattern), TogoVar (not reported) (Mitsuhashi et al., 2022), and ToMMo (not reported), and it is registered as “uncertain significance” in Clinvar. Her healthy sister also possessed a heterozygous p.P152L variant (Fig. 1, F and G). p.R306Q is an extremely rare variant in gnomAD (1 of 1,611,754 alleles in non-Finnish European adult population), ToMMo (not reported), Bravo (not reported), and TogoVar (not reported), and it occurred de novo. After subtracting de novo variants, trio-based WES strongly supported a biological relationship between the patient and her father and mother (Table S2). There were no other candidate mutations or substitutions that could cause IEIs (Table S2). Then, at the age of 2 years, the patient began treatment with the TNF-blocking agent etanercept, which led to the complete resolution of her symptoms (Fig. 1 A). Now, she has achieved steroid-free remission while on twice-weekly etanercept therapy and has started to catch up physically (Fig. 1 B).

**Table 1.** Pathogenic variants in the *OTULIN* gene of ORAS patients

	CDS position	AA alteration	Software prediction				Reference
			SIFT	PolyPhen-2	CADD	Mutation Taster	
Maternal allele	c.455C>T	p.P152L	0.21	0.002	21	Benign	This paper
de novo	c.917G>A	p.R306Q	0	0.999	32	Deleterious	This paper
ORAS	c.815T>C	p.L272P	0.03	0.999	29.2	Deleterious	Damgaard et al., 2016; Zhou et al., 2016
ORAS	c.731A>G	p.Y244C	0	0.925	26.6	Deleterious	Zhou et al., 2016
ORAS	c.841G>A	p.G281R	0	0.999	31	Deleterious	Damgaard et al., 2019
Atypical ORAS	c.258G>A	p.M86I	0.5	0.191	23.2	?	Zinngrebe et al., 2022
Atypical ORAS	c.500G>C	p.W167S	0	1	31	Deleterious	Zinngrebe et al., 2022

CDS, coding sequence; AA, amino acid; SIFT, Sorting Intolerant From Tolerant (<0.05 = deleterious); PolyPhen-2, Polymorphism Phenotyping v2 (>0.85 is “probably damaging” and <0.15 is “benign”); CADD, Combined Annotation Dependent Depletion (raw values have relative meaning, with higher values indicating a higher likelihood of deleterious effects).

### Patient-derived cells show increased linear ubiquitination

**Figure 2.**
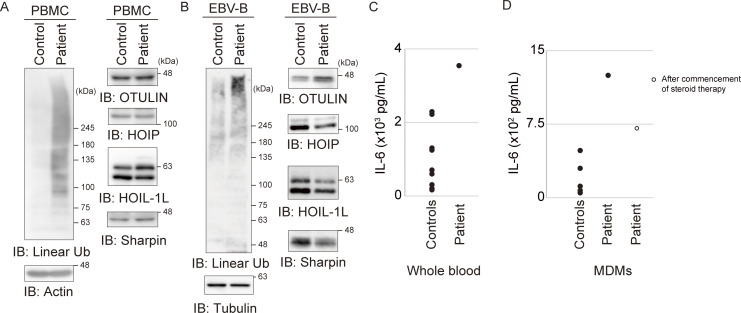
**Patient-derived cells show enhanced linear ubiquitination without any reduction in the amount of OULIN. (A and B)** The amounts of linear ubiquitin (linear Ub), OTULIN, and LUBAC components in patient-derived PBMCs (A) and patient-derived EBV-B cells (B) compared with those in healthy control cells. **(C)** IL-6 levels in the supernatant of IL-1β–stimulated whole blood samples from the patient and seven healthy controls. **(D)** IL-6 levels in culture supernatant from IL-1β–stimulated CD14-positive macrophages isolated from the patient before and after steroid treatment (compared with levels in seven healthy controls). IB, immunoblot. Source data are available for this figure: SourceData F2.

Several *OTULIN* variants are associated with an autoinflammatory disease called ORAS (Table S3) (Damgaard et al., 2016, 2019; Nabavi et al., 2019; Zhou et al., 2016; Zinngrebe et al., 2022). OTULIN is a DUB that specifically cleaves linear ubiquitin chains (Keusekotten et al., 2013; Rivkin et al., 2013). ORAS patients show a marked increase in the amount of linear ubiquitin chains due to a reduction in both the amount and DUB activity of OTULIN (Damgaard et al., 2016, 2019; Zhou et al., 2016; Zinngrebe et al., 2022). Immunoblot analysis revealed an increase in the amount of linear ubiquitin in peripheral blood mononuclear cells (PBMCs) and EBV-transformed B (EBV-B) cells derived from the patient, although the increase was much more evident in the latter (Fig. 2, A and B). This is likely because LUBAC-mediated linear ubiquitination plays a role in the activation of NF-κB by EBV-encoded LMP1 (Wang et al., 2017a) and is induced upon stimulation including TNF (Fuseya et al., 2020). In addition, increased production of IL-6 by patient-derived PBMCs and CD14-positive macrophages differentiated from PBMCs stimulated with IL-1β was observed (Fig. 2, C and D), confirming previous reports of some ORAS patients (Zhou et al., 2016). Although decreased expression of OTULIN protein is a common feature among ORAS patients (Damgaard et al., 2016, 2019; Zhou et al., 2016; Zinngrebe et al., 2022), the amount of OTULIN in the PBMCs and EBV-B cells from this patient was comparable with that in respective control cells (Fig. 2, A and B). Thus, the presence of augmented linear ubiquitination indicates that the patient was suffering from ORAS, although OTULIN levels were unaffected.

### The R306Q variant reduces OTULIN DUB activity

**Figure 3.**
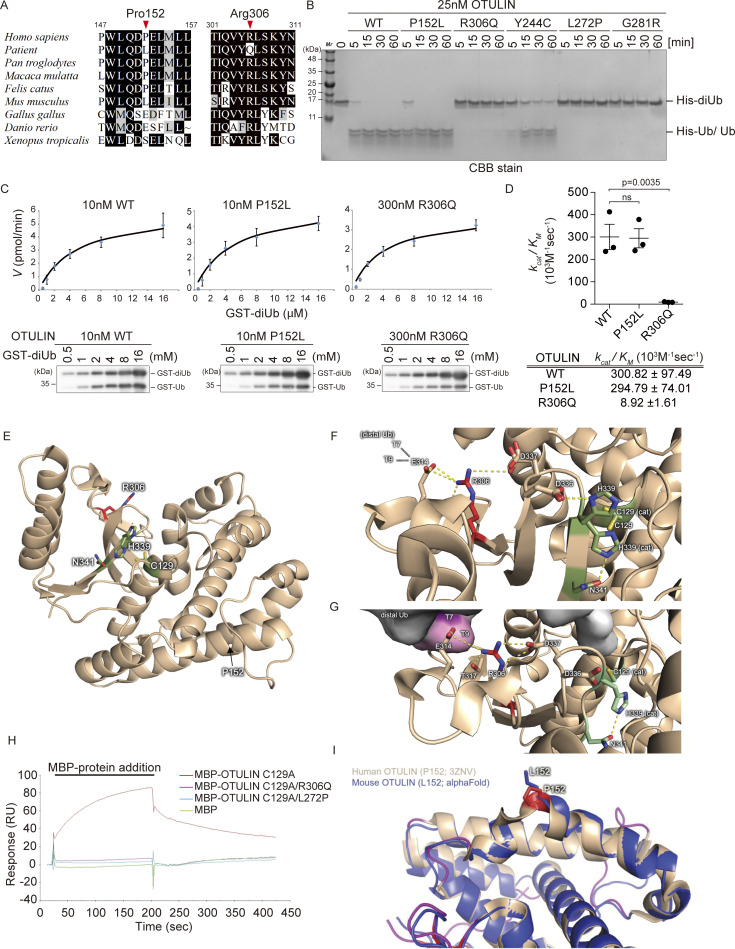
**Arg306 to Gln variant reduces the catalytic activity of OTULIN. (A)** Multiple sequence alignments of the residues surrounding Pro152 and Arg306 in OTULIN. Residues Pro152 and Arg306 are indicated by red arrowheads. **(B)** Hydrolysis of M1-linked His-diUb (2.5 µM) by recombinant OTULIN (WT and mutants; 25 nM) at the indicated time points, as visualized by CBB staining of SDS-PAGE gels. **(C and D)** Kinetic parameters determined by hydrolysis of M1-linked GST-diUb by 10 nM OTULIN^WT^ or OTULIN^P152L^, or 300 nM OTULIN^R306Q^ (C), along with rate constants (D). Data are performed with three independent experiments and expressed as the mean ± SEM. Statistical significance was assessed by one-way ANOVA. ns (not significant; P > 0.05) **(E)** Overall view of the OTU domain of OTULIN showing the position of Pro152 and Arg306 (PDB ID 3ZNV). **(F and G)** Role of Arg306 at the junction of the distal Ub binding site and in the catalytic core of OTULIN. While F shows the free structure of OTULIN (PDB ID 3ZNV), G displays the M1-linked diUb-bound structure (PDB ID 3ZNZ). **(H)** The affinity of recombinant catalytically inactive OTULIN mutants for M1-linked GST-diUb was measured by SPR. Catalytically inactive MBP-OTULIN^C129A^ (orange), MBP-OTULIN^C129A/R306Q^ (red), MBP-OTULIN^C129A/L272P^ (light blue), or control MBP protein (green) were used as analytes. **(I)** Visualization of the positioning of the sidechain of OTULIN residue 152 using an AlphaFold model of mouse OTULIN (blue ribbon; the Leu152 sidechain is represented by blue sticks) superimposed onto the crystal structure of human OTULIN (beige; the Pro152 sidechain is represented by red sticks; PDB ID 3ZNV). Source data are available for this figure: SourceData F3.

Two compound heterozygous *OTULIN* variants, p.P152L and p.R306Q, were identified in the patient. Analyses of the effect that these amino acid substitutions have on OTULIN function were conducted using the SIFT (Sim et al., 2012), Polyphen-2 (Adzhubei et al., 2010), CADD (Kircher et al., 2014), and Mutation Taster (Schwarz et al., 2014) databases. The results suggested that p.R306Q appears to be damaging, whereas p.P152L (which was inherited from her mother) is scored by in silico software as non-pathogenic (Table 1). In mice, the amino acid corresponding to P152 of human OTULIN is leucine, as is the case in the patient, whereas R306 is highly conserved evolutionarily (Fig. 3 A).

Next, to investigate the effect of these two amino acid substitutions on OTULIN function, we purified OTULIN variants from a bacterial expression system and evaluated their DUB activity by incubating them with linear di-ubiquitin (linear diUb) (Fig. 3, B and C). The P152L variant protein (OTULIN^P152L^) digested linear diUb as effectively as OTULIN WT; the enzymatic efficiency (*k*_cat_/K_M_) of OTULIN^P152L^ was 294.79 ± 74.01 (10^3^ M^−1^ sec^−1^), almost the same as that of the WT (300.82 ± 97.49 [10^3^ M^−1^ sec^−1^]) (Fig. 3 D). By contrast, the R306Q protein (OTULIN^R306Q^) showed heavily impaired DUB activity; the *k*_cat_/K_M_ of OTULIN^R306Q^ was 8.92 ± 1.61 (10^3^ M^−1^ sec^−1^) (Fig. 3 D). We also compared the DUB activity of OTULIN^R306Q^ with that of three previously reported ORAS variants: OTULIN^Y244C^, OTULIN^L272P^, and OTULIN^G281R^ (Damgaard et al., 2016, 2019; Zhou et al., 2016). We found that OTULIN^R306Q^ appeared to be less active than OTULIN^Y244C^ but more active than OTULIN^L272P^ and OTULIN^G281R^ (Fig. 3 B).

**Figure S1.**
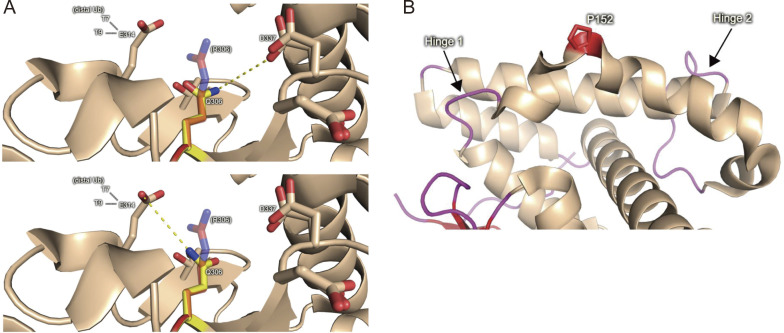
**The Arg306 to Gln mutation reduces the catalytic activity of OTULIN. (A)** Substitution of Arg306 with Gln weakens the Coulomb interactions by decreasing the strength of the participating charge, increasing the distance between residues, and disrupting hydrogen bonding. Two possible rotamers are shown. **(B)** Position of Pro152 in human OTULIN. Flexible hinge regions are indicated.

The crystal structures of the OTULIN OTU domain in both its free and linear diUb-bound forms have been reported (free form PDB ID 3ZNV; linear diUb-bound form PDB ID 3ZNZ) (Keusekotten et al., 2013). The positions of P152 and R306 and the catalytic triad (C129, H339, and N341) in the OTU domain of OTULIN (free form) are shown in Fig. 3 E. As shown in Fig. 3, E–G, R306 is located close to both the active center and the distal ubiquitin recognition site, while P152 is located far from both of these important sites. The side chain guanidinium group of R306 interacts electrostatically with E314, T317, and D337 within the two known OTULIN crystal structures (free and diUb-bound) (Fig. 3, F and G). E314 and T317 interact with the distal ubiquitin moiety of linear diUb, and their interaction with R306 appears to anchor those side chains to allow proper binding to the distal ubiquitin moiety (Fig. 3 G). Indeed, the variant in which E314 is replaced by arginine exhibits both reduced diUb binding affinity and reduced cleavage activity (Keusekotten et al., 2013). In addition, the electrostatic interactions between R306 and D337 may impact the positioning of the adjacent D336 residue. In the absence of substrate (linear diUb), D336 pulls the imidazole ring of H339, one of the active center (catalytic triad) residues, into a non-catalytic conformation (Keusekotten et al., 2013). In fact, the side chain distance (guanidium–carboxylate N–O) between R306 and D337 in the diUb-bound form is shorter than in the free form (3.1 Å versus 4.2 Å; Fig. 3, F and G). Therefore, R306 appears to contribute to the formation of the catalytically active orientation of H339 (Fig. 3 G, right panel). Thus, compared with previously reported amino acid residues that are involved directly in either ubiquitin binding (E314 and T317) or the structure of the active center (D336), R306 appears to have no direct effect on the DUB activity of OTULIN; indeed, R306 seems to play a central role in linking ubiquitin binding and active center formation in OTULIN, and its amino acid substitution to Q would be incompatible with those interactions, likely impairing OTULIN DUB activity greatly (Fig. S1 A). To confirm this, we purified recombinant OTULIN harboring the R306Q variant based on the DUB-deficient (C129A) variant to avoid cleavage of linear diUb and examined their binding to linear diUb using surface plasmon resonance (SPR) analysis. As predicted from the structural analysis, OTULIN^C129A/R306Q^ failed to interact with linear diUb to a similar extent as OTULIN^C129A/L272P^ (L272 resides in the ubiquitin-binding pocket and the L272P variant abolish linear ubiquitin binding; Damgaard et al., 2016) (Fig. 3 H).

By contrast, the P152L substitution had no structural or functional effect on the OTULIN OTU domain. In fact, comparison of the crystal structures of human OTULIN (PDB ID 3ZNV) and an AlphaFold model of mouse OTULIN, which harbors leucine as amino acid residue 152, indicated that P152 and L152 cause only minor structural changes (Fig. 3 I and Fig. S1 B). Taken together, the results of structural modeling and SPR analysis explain why OTULIN^P152L^ has the same DUB activity as OTULIN WT and why OTULIN^R306Q^ reduces DUB activity (Fig. 3 D).

### The R306Q variant in OTULIN augments TNF-mediated cell death

**Figure 4.**
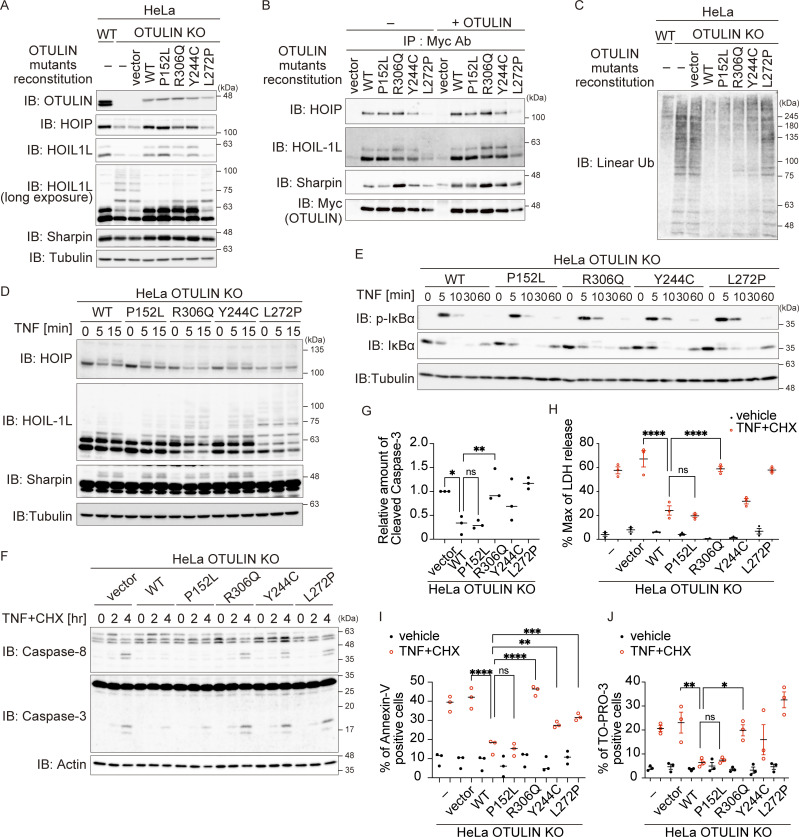
**The Arg306 to Gln variant accelerates TNF stimulation–dependent cell death. (A–G)** To analyze the intracellular function of OTULIN, HeLa OTULIN KO cells were reconstituted with an empty vector or with different myc-tagged OTULIN constructs, as indicated. **(A and C)** The amount of OTULIN, LUBAC components (A), and linear Ub (C) under unstimulated conditions. Data are representative of four (A) or five (C) independent experiments. Quantification and statistical analysis of the amount of linear ubiquitin is shown in Fig. S2 B. **(B)** Interaction between LUBAC components and OTULIN mutants. Myc-tagged immunoprecipitates were incubated with or without the recombinant OTULIN OTU domain and analyzed by immunoblotting (IB). Data are representative of two independent experiments. **(D)** Modification of LUBAC components in response to stimulation with FLAGHis-TNF (100 ng/ml). Data are representative of three independent experiments. **(E)** Immunoblot analysis of IκBα phosphorylation and degradation in the indicated cells treated with TNF (10 ng/ml) at the indicated times. Data are representative of three independent experiments. **(F and G)** Activated caspase proteins in the indicated cells in response to stimulation with TNF (10 ng/ml) and CHX (20 μg/ml). Densitometry analysis of cleaved caspase-3 was performed at 4 h after stimulation (G). Data are representative of three independent experiments and expressed as the mean ± SEM. **(H)** Death of the indicated cells following treatment with TNF (10 ng/ml) and CHX (20 µg/ml) was monitored in an LDH activity assay. Data are representative of three independent experiments and expressed as the mean ± SEM. **(I and J)** Percentage of Annexin-V and TO-PRO-3–positive cells in the indicated cell populations in response to stimulation with TNF (10 ng/ml) and CHX (20 µg/ml) for 14 h. Data are representative of three independent experiments and expressed as the mean ± SEM. **(G–J)** Statistical significance was assessed by one-way ANOVA. ns (not significant; P > 0.05), *P < 0.05, **P < 0.01, ***P < 0.001, or ****P < 0.0001. Source data are available for this figure: SourceData F4.

**Figure S2.**
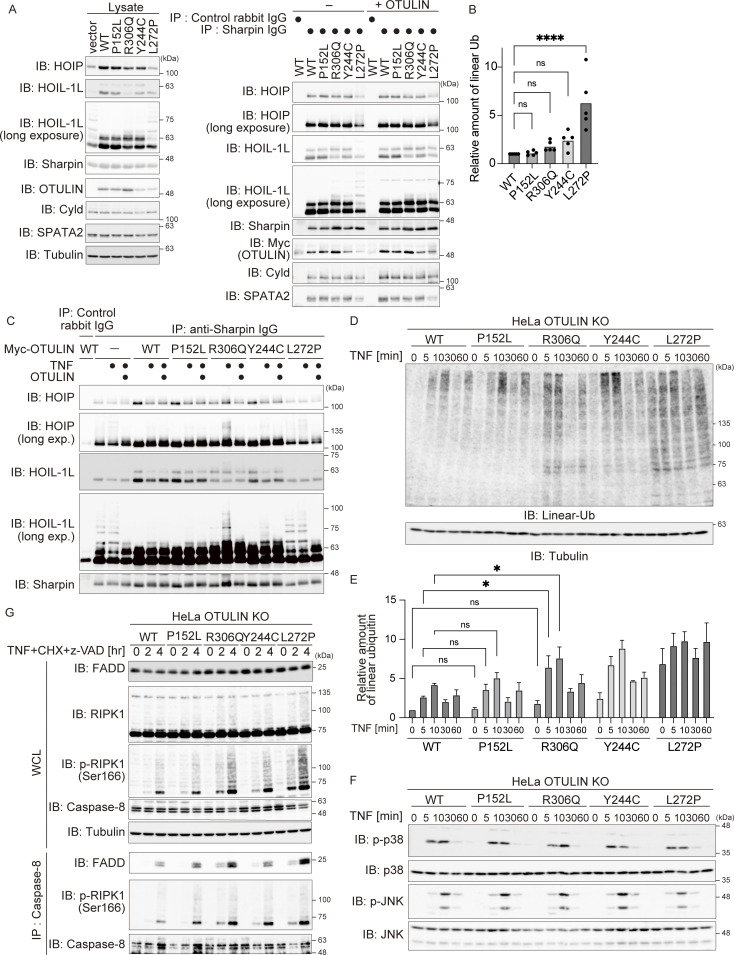
**The Arg306 to Gln mutation accelerates TNF stimulated modification of LUBAC. (A)** Analysis of the interaction between LUBAC components, OTULIN mutants, Cyld, and SPATA2. The indicated antibodies were used to co-precipitate LUBAC components and DUBs from HeLa OTULIN KO cells stably expressing the indicated OTULIN mutants. The immunoprecipitates were incubated at 37°C for 30 min with or without the recombinant OTULIN OTU-domain, and then analyzed by immunoblotting (IB). Data are representative of two independent experiments. **(B)** Relative amount of linear ubiquitin in the indicated HeLa cells. Five independent experiments were performed. Statistical significance was determined by one-way ANOVA. ns (not significant), or ****P < 0.0001. **(C)** Immunoblot analysis of LUBAC modifications in HeLa OTULIN KO cells stably expressing the indicated OTULIN mutants and treated for 10 min with TNF (10 ng/ml). Immunoprecipitates (obtained using the indicated antibodies) were also incubated at 37°C for 30 min with or without the recombinant OTULIN OTU-domain, and then analyzed by immunoblotting. Data are representative of two independent experiments. **(D and E)** Immunoblot analysis of linear ubiquitin in the same lysate shown in Fig. 4 E. Densitometry analysis of linear ubiquitin was performed. Data are representative of three independent experiments and expressed as the mean ± SEM. Statistical significance was assessed by two-way ANOVA. ns (not significant), or *P < 0.05. **(F)** Immunoblot analysis of the MAPK pathway in the same lysate is shown in Fig. 4 E. Data are representative of three independent experiments. **(G)** Purification of cell death–inducing Complex II (anti-caspase-8 immunoprecipitates) from the indicated cells. Cells were stimulated for the indicated times with TNF (10 ng/ml), CHX (20 µg/ml), or Z-VAD-FMK (10 µM). Data are representative of three independent experiments. Source data are available for this figure: SourceData FS2.

Next, to investigate the effect of OTULIN variants, we generated OTULIN-deficient HeLa cells using the CRISPR/Cas9 system and expressed OTULIN WT, patient-retained (OTULIN^P152L^ or OTULIN^R306Q^), or the previously reported ORAS variants (OTULIN^Y244C^ or OTULIN^L272P^) (Fig. 4 A). Overexpression of OTULIN inhibits TNF signaling (Keusekotten et al., 2013); therefore, we used a retrovirus expression system to express these mutants in OTULIN knock-out (KO) cells to evaluate its function. Although the amount of OTULIN introduced into cells was lower than that of endogenous OTULIN, we found that the reduction in the amount of LUBAC components in OTULIN-null HeLa cells, which is a characteristic of most cells that lack OTULIN (Keusekotten et al., 2013; Rivkin et al., 2013), was reversed by the introduction of OTULIN WT (Fig. 4 A). This indicates that this experimental system can be used to study OTULIN functions. The amounts of OTULIN^Y244C^ and OTULIN^L272P^ were lower than that of OTULIN WT, possibly because these variants affect protein stability as previously reported (Zhou et al., 2016). By contrast, the amounts of patient-derived OTULIN^P152L^ and OTULIN^R306Q^ were virtually the same as those of the WT (Fig. 4 A). The N-terminal region of OTULIN binds to the PUB domain of HOIP (Elliott et al., 2014; Schaeffer et al., 2014; Takiuchi et al., 2014); therefore, we confirmed that the patient-derived OTULIN variants bound to LUBAC as effectively as WT by performing coimmunoprecipitation experiments; the results were consistent with the fact that the P152 and R306 residues do not reside in the N-terminal region of OTULIN (Fig. 4 B and Fig. S2 A).

Auto-linear ubiquitination of LUBAC subunits, including HOIL-1L, is higher in cells with null or reduced OTULIN DUB activity, which results in attenuation of LUBAC activity (Fuseya et al., 2020; Heger et al., 2018). Indeed, the ladder-like modification of HOIL-1L in HeLa OTULIN KO cells and in cells expressing DUB activity-defective OTULIN^L272P^ was enhanced (Fig. 4 A). Ladder-like modification of HOIL-1L in cells expressing OTULIN^P152L^ was as weak as that in the WT, whereas an additional band of HOIL-1L, which migrated more slowly, was observed in cells expressing OTULIN^R306Q^, albeit weakly (Fig. 4 A; HOIL-1L long exposure). This additional and slowly migrating HOIL-1L band in OTULIN^R306Q^-expressing cells disappeared upon incubation with OTULIN, suggesting that it represents linear ubiquitinated HOIL-1L (Fig. 4 B and Fig. S2 A). The amount of linear ubiquitin in unstimulated cells expressing OTULIN^R306Q^ appeared to increase, albeit much less markedly than that in OTULIN KO cells (Fig. 4 C and Fig. S2 B).

TNF stimulation augmented the laddered HOIL-1L signal significantly in cells expressing OTULIN^R306Q^ when compared with those expressing the WT, confirming the attenuated LUBAC activity observed in OTULIN^R306Q^-expressing cells; this was not the case in OTULIN^P152L^-expressing cells (Fig. 4 D and Fig. S2, C–E). Because LUBAC-mediated linear ubiquitination plays crucial roles in NF-κB activation and inhibition of regulated cell death (Fuseya and Iwai, 2021; Peltzer and Walczak, 2019; van Loo and Bertrand, 2023), we examined the effect of the p.R306Q and p.P152L variants on TNF-mediated activation of NF-κB. The OTULIN^R306Q^ or OTULIN^P152L^ variants did not overtly affect phosphorylation and degradation of IκBα, which are the hallmarks of NF-κB activation, or phosphorylation of p38 and JNK; this was also the case for OTULIN^L272P^ and OTULIN^Y244C^, both of which are mutants known to induce ORAS (Fig. 4 E and Fig. S2 F). This suggests that impaired OTULIN DUB activity does not overtly affect TNF-mediated signaling (including NF-κB activation), at least in our experimental setting. Next, we treated cells with TNF and cycloheximide (CHX) to evaluate TNF-mediated cell death. TNF+CHX augmented cleavage of caspase-8 and caspase-3, release of lactate dehydrogenase (LDH), and Annexin-V and TO-Pro-3 positivity in OTULIN^R306Q^-expressing cells, but not in OTULIN^P152L^-expressing cells (Fig. 4, F–J), suggesting that the p.R306Q variant provides reduced protection from cell death. This was confirmed by the finding that both phosphorylation of RIPK1 (p-S166), which sensitizes cells to TNF-induced death, and Complex II formation increased in OTULIN^R306Q^- but not in OTULIN^P152L^-expressing cells (Fig. S2 G). It is worth noting that activation of caspases and formation of Complex II were also observed in cells expressing the ORAS-inducing p.Y244C and p.L272P variants (Fig. 4 F and Fig. S2 G). Taken together, the data suggest that OTULIN^R306Q^ behaves similarly to other ORAS-inducing OTULIN mutants, whereas OTULIN^P152L^ is more similar to the WT, as predicted by structural modeling.

### Patient-derived cells are more susceptible to regulated cell death

**Figure S3.**
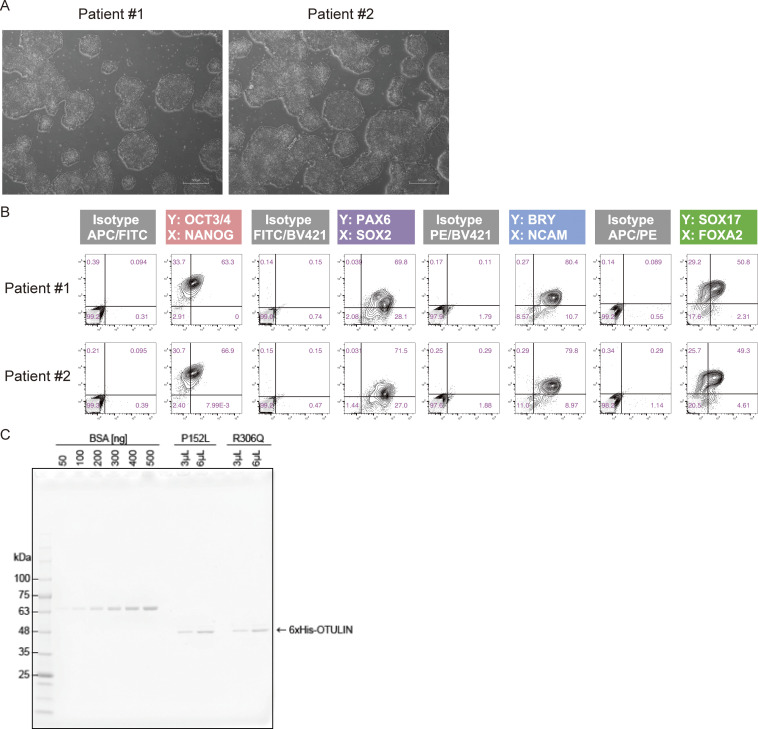
**Analysis of patient-derived iPS cells. (A)** Representative images of patient-derived iPS colonies. All images were captured at 4× magnification. Scale bars: 500 µm. **(B)** Flow cytometry analysis of the in vitro differentiation capacity of patient-derived lines. **(C)** Isotope-labeled recombinant OTULIN mutants. The His-tag purified proteins were visualized, along with a BSA standard, by CBB staining of SDS-PAGE gels. Source data are available for this figure: SourceData FS3.

**Figure 5.**
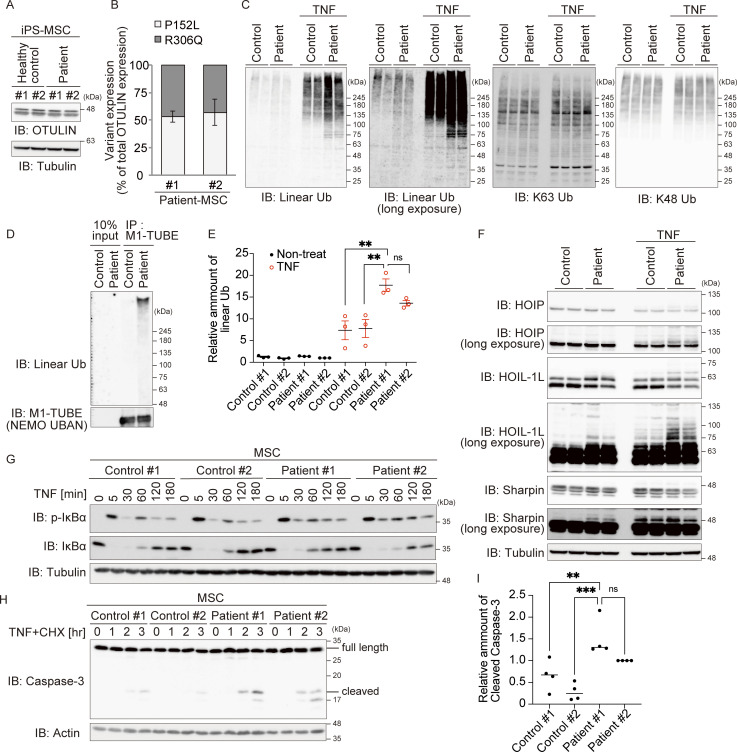
**Patient-derived cells show impaired protection against cell death. (A)** The amount of OTULIN in MSCs differentiated from healthy control or patient iPS cells. **(B)** The ratio of p.P152L to p.R306Q variant proteins in the patient’s MSCs was evaluated by MS/MS analysis. Isotope-labeled OTULIN variant proteins (as shown in Fig. S3 C) were added to lysates as an evaluation control. Bars represent the mean ± SEM (*n* = 3). **(C and E)** Immunoblot (IB) analysis of specific ubiquitin chains in MSCs treated (or not) with TNF (10 ng/ml) for 10 min. Data are representative of three independent experiments. Quantification and statistical analysis of the amount of linear ubiquitin detected in C is shown in E. Statistical significance was assessed by one-way ANOVA. ns (not significant; P > 0.05), or **P < 0.01. **(D)** Purification of linear ubiquitin from unstimulated MSC lysates using M1-TUBE (Halo-NEMO-UBAN). **(F)** Immunoblot analysis of LUBAC components in MSCs treated (or not) with TNF (10 ng/ml) for 10 min. Data are representative of three independent experiments. **(G)** Immunoblot analysis of IκBα phosphorylation and degradation in MSCs treated with TNF (10 ng/ml) for the indicated times. Data are representative of three independent experiments. **(H and I)** Activated caspase proteins in MSCs stimulated with TNF (10 ng/ml) and CHX (20 µg/ml). Densitometry analysis of cleaved caspase-3 at 3 h after stimulation with TNF+CHX (I). Data are representative of three independent experiments and expressed as the mean ± SEM. Statistical significance was assessed by one-way ANOVA. ns (not significant; P > 0.05), **P < 0.01, or ***P < 0.001. Source data are available for this figure: SourceData F5.

Because patient-derived primary fibroblasts were unavailable, we established two clones of patient-derived induced pluripotent stem cells (iPSCs) with sufficient differentiation potential for further analyses (Fig. S3, A and B) (Shiba et al., 2019). Because the patient responded well to an anti-TNF agent (Fig. 1 A and Clinical description), we next evaluated the effect of the OTULIN variants under conditions of TNF stimulation. To do this, iPSCs were differentiated into mesenchymal stem cells (MSCs), which exhibit characteristics very similar to those of fibroblasts and respond to TNF stimulation (Soundararajan and Kannan, 2018; Ugurlu and Karaoz, 2020). First, we examined the amount of OTULIN in MSCs and found that expression in patient-derived MSCs was almost equal to that in control MSCs (Fig. 5 A). Next, we investigated the OTULIN^P152L^ to OTULIN^R306Q^ ratio by mass spectrometry using stable isotope-labeled recombinant OTULIN variants as indicators (Fig. S3 C). We found no significant difference between the expression of OTULIN^P152L^ and that of OTULIN^R306Q^ (Fig. 5 B), indicating that the p.R306Q variant does not overtly affect the amount of the protein and that approximately half of the OTULIN in patient-derived cells is the pathogenic mutant.

**Figure S4.**
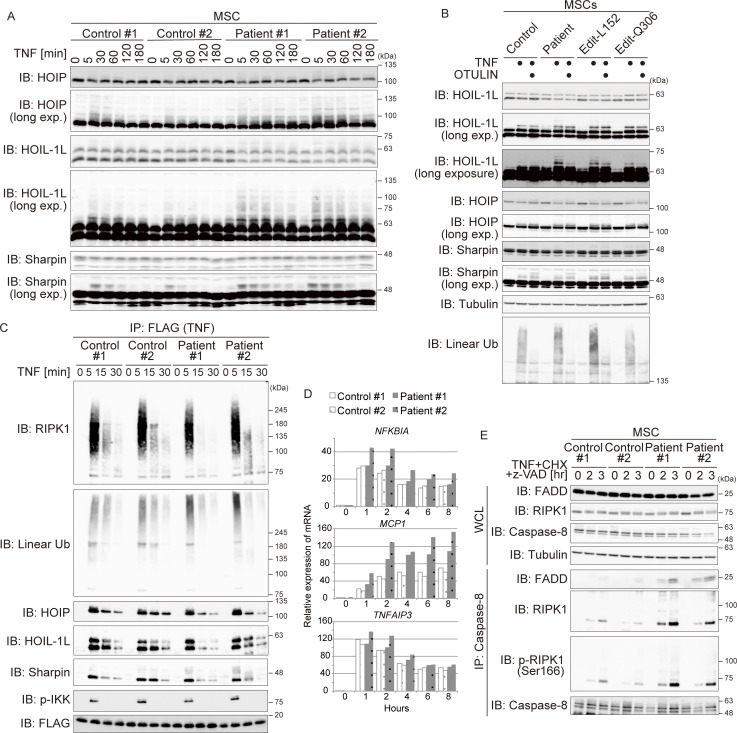
**Analysis of patient iPS-derived MSCs. (A)** Immunoblot (IB) analysis of LUBAC components in MSCs treated with TNF (10 ng/ml) for the indicated times. Data are representative of three independent experiments. **(B)** Immunoblot analysis of modified LUBAC in MSCs treated (or not) for 10 min with TNF (10 ng/ml). After treatment with TNF, MSCs were harvested and lysed. Cell lysates were incubated at 37°C for 30 min in the presence/absence of the recombinant OTULIN OTU-domain and analyzed by immunoblotting. Data are representative of two independent experiments. **(C)** Purification of TNFR Complex I (anti-FLAG [TNF] immunoprecipitates [IP]) from MSCs treated with FLAGHis-TNF (100 ng/ml) at the indicated times. Data are representative of three independent experiments. **(D)** Relative expression of mRNA encoding several NF-κB targets (IκBα, A20, and CCL2) was measured by qPCR. The mRNA was isolated from MSCs treated with TNF (10 ng/ml) for the indicated times. **(E)** Purification of cell death–inducing Complex II (anti-caspase-8 immunoprecipitates) from MSCs. Cells were stimulated for the indicated times with TNF (10 ng/ml), CHX (20 µg/ml), and Z-VAD-FMK (10 µM). Data are representative of three independent experiments. Source data are available for this figure: SourceData FS4.

Next, we examined the amount of linear ubiquitin in MSCs. Since the amount of linear chain in unstimulated cells is very small (Fig. 5 C), as was the case with HeLa cells (Fig. 4 C), we enriched linear ubiquitin using M1-TUBE and found that the amount of total linear ubiquitin was higher in patient-derived MSCs than in control MSCs (Fig. 5 D). This confirmed that the amount of linear ubiquitin chains in patient-derived MSCs was significantly higher than that in control MSCs under conditions of TNF stimulation (Fig. 5, C and E). By contrast, there was no difference in the amount of K63 and K48 ubiquitin chains in patients and control MSCs (Fig. 5 C). Auto-linear ubiquitination of LUBAC subunits, including HOIL-1L, underlies suppression of LUBAC function, including protection from cell death (Fuseya et al., 2020; Heger et al., 2018). Although the laddered-signal of HOIL-1L was detected in patient-derived MSCs in the absence of TNF (albeit very weakly) (Fig. 5 F), we found that TNF stimulation increased the laddered-signal of HOIL-1L markedly, as well as that of HOIP, in patient-derived MSCs when compared with control cells (Fig. 5 F and Fig. S4 A). These slower migrating signals in MSCs represent linear ubiquitinated HOIL-1L because they disappeared when the lysates were incubated with OTULIN, implying suppression of LUBAC function in patient-derived MSCs (Fig. S4 B). Collectively, the data suggest that the amount of total cellular linear ubiquitin in patient-derived MSCs is increased, as described previously in cells derived from ORAS patients (Damgaard et al., 2016, 2019; Zhou et al., 2016).

Next, we evaluated TNF-dependent NF-κB activation in MSCs, as well as protection from cell death. There was no clear difference in phosphorylation and degradation of IκBα, or in the recruitment of LUBAC to the TNF receptor complex, between patient and control MSCs (Fig. 5 G and Fig. S4 C). Also, there was no reduction in the expression of mRNAs encoding NF-κB target genes in TNF-stimulated MSCs (Fig. S4 D), confirming that impairment of OTULIN DUB activity does not affect TNF-mediated NF-κB activation, as observed in HeLa cells (Fig. 4 E). However, when stimulated with TNF+CHX, cleavage of caspase-3, which reflects activation of apoptosis, was more pronounced in patient-derived MSCs than in control MSCs (Fig. 5, H and I). Consistent with these results, formation of Complex II increased in both clones of patient-derived MSCs (Fig. S4 E), indicating that protection from TNF-induced cell death is impaired in patient-derived MSCs, as was the case for HeLa cells expressing OTULIN^R306Q^ or OTULIN^L272P^ (Fig. 4). Collectively, the data suggest that NF-κB activation and protection from cell death in patient-derived cells is similar to that in cells expressing ORAS-oriented OTULIN mutants, even though only half of the total amount of OTULIN in patient-derived cells is pathogenic.

### OTULIN^R306Q^ may trigger ORAS by acting as a dominant-negative mutant

**Figure 6.**
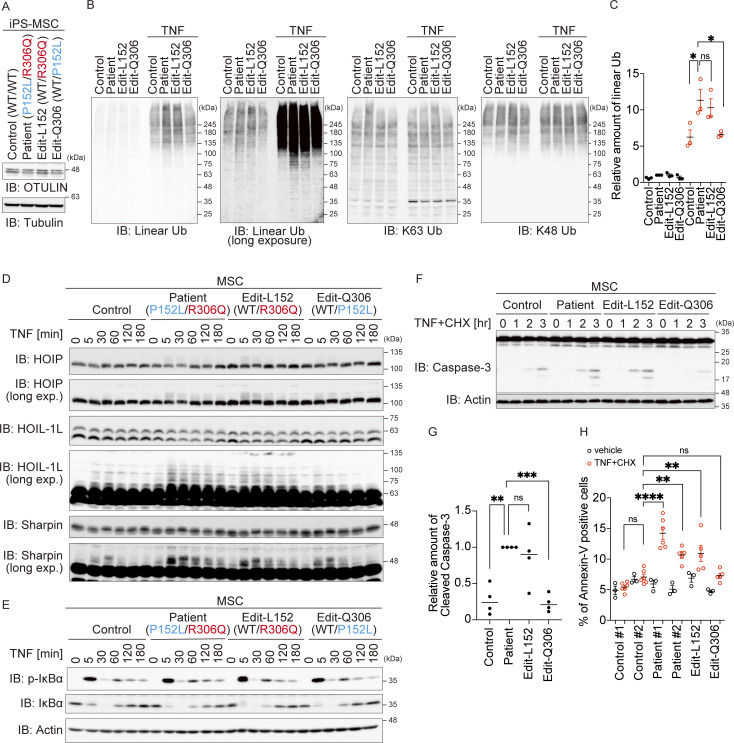
**Only the de novo p.R306Q heterozygous variant in the patient augments TNF-dependent cell death. (A)** Amount of OTULIN in MSCs differentiated from healthy controls, patient-derived iPS, and base-edited L152 or Q306 cells. Data are representative of three independent experiments. **(B and C)** Immunoblot (IB) analysis of specific ubiquitin chains in MSCs treated (or not) with TNF (10 ng/ml) for 10 min. Data are representative of three independent experiments. Quantification and statistical analysis of the amount of linear ubiquitin detected in B was shown in C. Statistical significance was assessed by one-way ANOVA. ns (not significant), or *P < 0.05. **(D and E)** Immunoblot analysis of LUBAC components (D), and of IκBα phosphorylation and degradation (E), in MSCs treated with TNF (10 ng/ml) for the indicated times. Data are representative of three independent experiments. **(F and G)** Activated caspase proteins in MSCs in response to stimulation with TNF (10 ng/ml) and CHX (20 µg/ml). Densitometry analysis of cleaved caspase-3 was performed at 3 h after stimulation with TNF+CHX (G). Data are representative of three independent experiments and are expressed as the mean ± SEM. **(H)** Flow cytometry analysis of Annexin-V–positive cells. MSCs were treated (or not) with TNF and CHX for 8 h. Four or six independent experiments, without or with TNF+CHX, respectively. Data are expressed as the mean ± SEM. **(G and H)** Statistical significance was assessed by one-way ANOVA. ns (not significant; P > 0.05), **P < 0.01, ***P < 0.001, or ****P < 0.0001. Source data are available for this figure: SourceData F6.

ORAS is caused by autosomal recessive traits (homozygous or compound heterozygous variants in *OTULIN*), and all *OTULIN* variants reported to date have attenuated DUB activity (Damgaard et al., 2016, 2019; Nabavi et al., 2019; Zhou et al., 2016; Zinngrebe et al., 2022). However, we found that OTULIN^P152L^ appears to be equivalent to WT, while OTULIN^R306Q^ is pathogenic. This raises the possibility that the p.R306Q heterozygous variant, which occurred de novo in the patient (Fig. 1 F), could cause ORAS in an autosomal-dominant fashion. To examine this hypothesis, we edited the *OTULIN* gene in patient-derived iPSCs using base editing technology based on the CRISPR/Cas9 system to establish cells expressing OTULIN^R306Q^ and WT (Edit-L152: WT/R306Q), or OTULIN^P152L^ and WT (Edit-Q306: WT/P152L) (Koblan et al., 2018). Then, we differentiated them into MSCs. Replacement of either variant with WT did not alter the expression of OTULIN protein in MSCs (Fig. 6 A), which implies that both p.R306Q and p.P152L variations do not affect the stability of OTULIN. We found that the amount of total intracellular linear ubiquitin was significantly increased in MSCs expressing OTULIN^R306Q^ (patient and Edit-L152 cells) upon TNF treatment, although there was no difference in the amount of K48 or K63 chains (Fig. 6, B and C). To determine whether the expression of OTULIN^R306Q^ is sufficient to induce autolinear ubiquitination of LUBAC subunits, we stimulated MSCs with TNF. TNF-induced auto-linear ubiquitination of LUBAC subunits was augmented in the patient’s cells and Edit-L152 cells (Fig. 6 D). This strongly indicates that the heterozygous p.R306Q variant is sufficient to increase not only the total amount of intracellular linear ubiquitin but also that of auto-linear ubiquitination of LUBAC subunits. We then examined the impairment of LUBAC function in heterozygous OTULIN^R306Q^-expressing cells. We found no marked difference in TNF-mediated phosphorylation or degradation of IκBα in any of the four MSC clones (Fig. 6 E); however, TNF-dependent cell death, as evaluated by cleavage of caspase-3 activation and Annexin-V staining, revealed that Edit-L152 (WT/R306Q) cells were as sensitive to TNF-mediated cell death as patient-derived MSCs and more sensitive than control or Edit-Q306 (WT/P152L) cells (Fig. 6, F–H).

**Figure S5.**
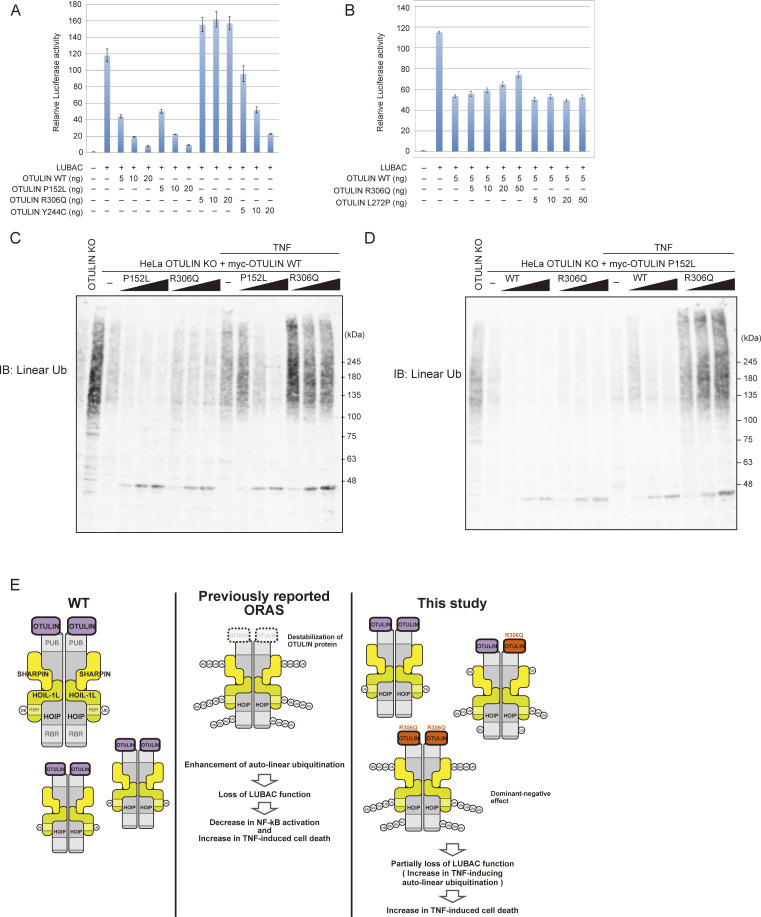
**The Arg306 to Gln variant has a dominant-negative effect. (A and B)** Luciferase activity of each OTULIN mutant (A), and coexpression with OTULIN WT and OTULIN^R306Q^ or OTULIN^L272P^ (B). HEK293T cells were transfected with the indicated amounts of plasmid, and luciferase activity was measured. Error bars represent the standard deviation from triplicate experiments. **(C and D)** The amount of linear ubiquitin in response to stimulation by TNF (10 ng/ml) for 10 min. IB, immunoblot. C shows myc-OTULIN WT expressing cells, and D shows myc-OTULIN P152L expressing cells. **(E)** Schematic showing the cellular effects of OTULIN mutations. Source data are available for this figure: SourceData FS5.

Finally, we examined the mechanism by which the heterozygous p.R306Q variant provokes ORAS. Because exogenous OTULIN suppresses LUBAC-mediated NF-κB activation (Keusekotten et al., 2013; Takiuchi et al., 2014), we first performed an NF-κB reporter assay after introducing expression plasmids carrying mutant or OTULIN WT, together with plasmids carrying the three LUBAC subunits, into HEK293T cells (Fig. S5 A). Although OTULIN^P152L^ suppressed LUBAC-mediated NF-κB activation at a level equivalent to that of WT, OTULIN^R306Q^ augmented NF-κB activation; in addition, pathogenic OTULIN^Y244C^ suppressed NF-κB activation, albeit less efficiently than the WT protein (Fig. S5 A). Because HEK293T cells express endogenous OTULIN, we suspected that enforced but stable expression of DUB-defective OTULIN^R306Q^ may suppress the function of endogenous OTULIN in a dominant-negative manner, thereby augmenting LUBAC-mediated NF-κB activation. Indeed, the introduction of OTULIN^R306Q^ counteracted suppression of LUBAC-mediated NF-κB activation by exogenous OTULIN WT in a dose-dependent manner, whereas OTULIN^L272P^, a known ORAS-provoking mutant, did not (Fig. S5 B). These data implied that OTULIN^R306Q^ suppresses OTULIN function in a dominant-negative manner.

**Figure 7.**
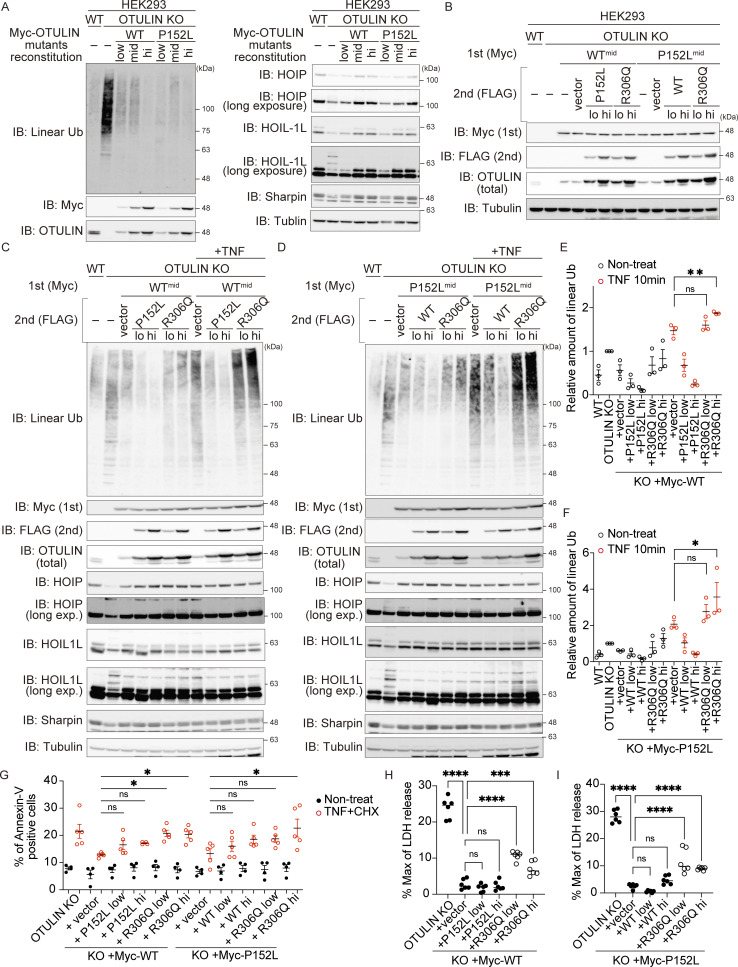
**The Arg306 to Gln variant acts in a dominant-negative manner. (A)** HEK293 OTULIN KO cells were reconstituted with the myc-tagged OTULIN WT or P152L constructs, as indicated. Low, mid, and high represent the amount of retrovirus infected to OTULIN KO cells. IB, immunoblot. **(B)** To analyze the dominant-negative effect of the OTULIN R306Q variant, HEK293 OTULIN KO cells expressing OTULIN WT^mid^ or P152L^mid^ were reconstituted with the FLAG-tagged OTULIN constructs, as indicated. **(C–F)** Amount of linear ubiquitin, and modification of LUBAC components, in response to stimulation with TNF (10 ng/ml) for 10 min. Quantification and statistical analysis of the amount of linear ubiquitin detected in C or D were shown in E and F, respectively. Data are representative of three independent experiments and are expressed as the means ± SEM. **(G)** Percentage of Annexin-V–positive cells in the indicated cells in response to stimulation with TNF (10 ng/ml) and CHX (20 µg/ml) for 4 h. Data are expressed as the mean ± SEM. **(H and I)** Death of the indicated cells following treatment with TNF (10 ng/ml) and CHX (20 µg/ml) for 6 h was monitored in an LDH activity assay. Four or five independent experiments were performed, without or with TNF+CHX, respectively. Data are expressed as the mean ± SEM. **(E–I)** Statistical significance was assessed by one-way ANOVA. ns (not significant; P > 0.05), *P < 0.05, **P < 0.01, ***P < 0.001, or ****P < 0.0001. Source data are available for this figure: SourceData F7.

To exert the dominant negative function, the p.R306Q variant should counteract the reduction of linear chains provoked by OTULIN WT or the p.P152L variant in a dose-dependent manner. To examine it, we introduced OTULIN WT or the OTULIN^P152L^ variant into HEK293 cells lacking OTULIN, which were generated using the CRISPR/Cas9 system (Fig. 7 A). Introduction of the OTULIN^P152L^ variant effectively reduced the amount of the linear chain (which was increased by the loss of OTULIN) as effectively as OTULIN WT when expressed at a level comparable with that of endogenous OTULIN (Fig. 7 A); this confirmed that the OTULIN^P152L^ variant is equivalent to the WT. We then introduced different amounts of the OTULIN^R306Q^ variant into OTULIN-null HEK293 cells expressing OTULIN WT or OTULIN^P152L^ (Fig. 7 B). As shown in Fig. 7 B, OTULIN^R306Q^ reversed the decrease of linear chains mediated by OTULIN WT or OTULIN^P152L^ in a dose-dependent manner (Fig. 7, C–F). We also confirmed the reversal of the OTULIN WT- or OTULIN^P152L^-mediated reduction of linear chains by the OTULIN^R306Q^ variant in HeLa cells (Fig. S5, C and D). These results indicate that the p.R306Q variant possesses the dominant-negative function to counteract the cleavage of linear chains by the p.P152L variant as well as by OTULIN WT. To further evaluate if the dominant-negative function of the p.R306Q variant underlies the augmented cell death, we treated OTULIN-null HEK293 cells expressing OTULIN WT and/or variants with TNF+CHX. Introduction of the OTULIN^R306Q^ variant significantly reversed the OTULIN WT- or OTULIN^P152L^-mediated rescue of death in OTULIN-null HEK293 cells (Fig. 7, G–I), which confirms that the dominant-negative function of the OTULIN p.R306Q variant is causative to augmented TNF-mediated cell death, which is the characteristic feature of patient-derived MSCs.

## Discussion

Here, we report the case of a girl with neonatal-onset severe autoinflammation, neutrophilic dermatitis, respiratory distress, and growth failure (confirmed to be ORAS), who was treated successfully with the anti-TNF agent, etanercept. ORAS can be caused by reduced OTULIN activity resulting from biallelic *OTULIN* variants (either homozygous or compound heterozygous variants) (Damgaard et al., 2016, 2019; Nabavi et al., 2019; Zhou et al., 2016; Zinngrebe et al., 2022). This ORAS patient possessed two variants in different alleles: p.P152L and p.R306Q. p.P152L is reported as rs547080675 in the dbSNP and is registered in gnomAD, whereas the pathogenic p.R306Q is an extremely rare variant in these databases; in only one adult categorized aged 50–55 years this heterozygous variant has been reported, although both germline and somatic variants are stored in gnomAD (Karczewski et al., 2020; Mitsuhashi et al., 2022; Sherry et al., 2001); we suspected that it might be a somatic variant because a somatic heterozygous *OTULIN* p.R306Q variant was identified by WES in a cancer specimen from a single patient (Giannakis et al., 2016). Therefore, it is unlikely that a germline p.R306Q variant has been reported. The p.R306Q variant, which is a de novo variant, exhibited attenuated DUB activity, whereas the activity of the p.P152L variant was comparable with that of WT (Fig. 3, C and D). More importantly, base-edited MSCs expressing OTULIN^R306Q^ and OTULIN WT were as sensitive to TNF-mediated cell death (a characteristic feature of ORAS) as the original patient-derived MSCs (Fig. 6, F–J). Since the amounts of OTULIN^P152L^ and OTULIN^R306Q^ in patient-derived MSCs were almost identical (Fig. 5 B), our data strongly suggest that a dominant-negative OTULIN mutant can trigger ORAS in an autosomal-dominant manner.

LUBAC comprises three subunits: the catalytic HOIP and the accessory subunits HOIL-1L and SHARPIN (Kirisako et al., 2006; Tokunaga et al., 2011); however, gel filtration analyses show that each LUBAC complex contains more than two of each subunit (Kirisako et al., 2006). Recently, we found that loss of ubiquitin ligase activity by the accessory subunit HOIL-1L augments LUBAC function by inhibiting auto-linear ubiquitination of LUBAC. Introduction of even one *HOIL-1L* (*RBCK1*) mutant allele lacking ligase activity is sufficient to augment LUBAC function in mice (Fuseya et al., 2020). This implies that a reduction in, rather than complete loss of, HOIL-1L ligase activity can suppress auto-linear ubiquitination. Since OTULIN interacts with LUBAC via the N-terminal PUB domain of HOIP (Elliott et al., 2014; Schaeffer et al., 2014; Takiuchi et al., 2014), at least two OTULIN proteins exist within one LUBAC complex. OTULIN maintains LUBAC function by removing auto-linear ubiquitin chains from LUBAC (Heger et al., 2018). Since we observed that both OTULIN variants (p.R306Q and p.P152L) can bind to HOIP and that linear ubiquitination of LUBAC subunits is augmented in patient-derived MSCs (Fig. 4 B and Fig. 6 D), it is plausible that the p.R306Q variant exerts its dominant-negative function in the LUBAC complex to enhance auto-linear ubiquitination of LUBAC, which results in the suppression of LUBAC function to augment TNF-mediated cell death. Therefore, we suspect that the heterozygous *OTULIN* variant alone can cause ORAS in a dominant-negative fashion.

Among the few ORAS patients reported to date, the majority harbor homozygous *OTULIN* variants. With the exception of one patient with a p.Y244C homozygous variant whose disease onset was at 4.5 mo of age, those with homozygous variants exhibited severe symptoms within the first month of life (Table S3) (Damgaard et al., 2016, 2019; Nabavi et al., 2019; Zhou et al., 2016). Although both p.Y244C and p.L272P variants cause autosomal recessive ORAS, p.Y244C exhibited greater DUB activity than p.L272P (Fig. 3 B). Reflecting these residual activities, the patient with p.Y244C was managed with the anti-IL-1β agent, Anakinra, whereas the patient with p.L272P patient could not be managed with Anakinra; rather, they required treatment with the anti-TNF agent infliximab (Damgaard et al., 2016; Zhou et al., 2016). Our patient suffered from ORAS since birth, although the symptoms were less severe than those reported for cases with autosomal recessive ORAS of neonatal onset, including those with the p.L272P variant. This is likely because in our patient the symptoms were managed in part by high-dose of prednisolone therapy (see Clinical description). A recent study reported that a patient with compound heterozygous variants in OTULIN exhibited symptoms during childhood, which responded well to prednisolone (Zinngrebe et al., 2022). This patient had two OTULIN variants, p.M86I and p.W167S, both of which resulted in mildly impaired linear ubiquitin and DUB activity (Zinngrebe et al., 2022). The p.M86I and p.W167S variants sensitized fibroblasts to TNF-mediated cell death only mildly, implying that OTULIN DUB activity in our patient appeared to be affected more severely than that in patients with compound heterozygous variants. Our patient exhibited symptoms of ORAS even though only the p.R306Q variant is pathogenic. Since HeLa cells expressing OTULIN^L272P^ show much higher levels of TNF-dependent auto-linear ubiquitination of LUBAC subunits than cells expressing OTULIN^Y244C^ or OTULIN^R306Q^ (Fig. 4 D), the severity of ORAS symptoms appears to correlate with residual OTULIN function rather than with the mode of inheritance.

We also found augmented TNF-induced cell death of MSCs derived from our patient, whereas TNF-mediated NF-κB activation was not affected (Fig. 5 F and Fig. S5 B). However, fibroblastic cells from other neonatal-onset cases of autosomal recessive ORAS (Damgaard et al., 2016, 2019; Zhou et al., 2016) or fibroblasts from mice expressing the C129A mutant, which completely lacks DUB activity (Heger et al., 2018), showed attenuated TNF-mediated NF-κB activation. The mechanism underlying this difference is unknown. However, there were some differences between our patient and other autosomal recessive ORAS patients and DUB-null mice. For example, the amount of LUBAC subunits in unstimulated patient-derived MSCs was comparable with that in healthy controls (Fig. 5 F), whereas fibroblasts derived from autosomal recessive ORAS patients with severe phenotypes and mice expressing the C129A mutant exhibited profound reductions in the amount of LUBAC subunits without any stimulation. We confirmed that the amount of LUBAC was indeed reduced in OTULIN^L272P^-expressing HeLa cells (Fig. 4 A). The reduction in the amount of LUBAC subunits in OTULIN^C129A^-expressing cells was reversed by an inactive HOIP catalytic variant (Heger et al., 2018), indicating that auto-linear ubiquitination underlies the reduction in the amount of LUBAC subunits. Considering the very low levels of auto-linear ubiquitinated LUBAC subunits and linear ubiquitin chains in unstimulated patient-derived MSCs (Fig. 5 C), it appears plausible that auto-linear ubiquitination of LUBAC in unstimulated cells, i.e., the reduction of LUBAC activity, is much milder in our patient than in other cases of ORAS or in OTULIN^C129A^-expressing mice because of residual OTULIN function in the patient. Thus, a sufficient amount of unmodified LUBAC remains in unstimulated patient-derived cells, and once stimulated with TNF enough LUBAC can be recruited to Complex I for adequate activation of NF-κB (Fig. 5 G), suggesting that reduced activation of NF-κB is not a common feature of ORAS patients. In patient-derived MSCs, however, TNF-dependent cell death is augmented. This type of cell death is suppressed by the linear ubiquitination of RIPK1 and other components within Complex I (Tu et al., 2021). Upon recruitment to Complex I, prolonged auto-linear ubiquitination of LUBAC occurs (Fig. 5 E), resulting in attenuated LUBAC function and reduced linear ubiquitination of Complex I proteins, as well as formation of cell death–inducing Complex II (Fig. 5 I), in patient-derived cells. As mentioned before, augmented TNF-induced cell death is a common characteristic of fibroblastic cells with severely impaired OTULIN activity, and enhanced cell death acts as a proinflammatory factor via the release of DAMPs and other substances (Luo et al., 2022; Wang et al., 2017b). Considering that anti-TNF therapy is highly effective in ORAS cases (Damgaard et al., 2016, 2019; Zhou et al., 2016), including the current patient, we speculate that enhanced cell death due to decreased OTULIN activity plays a crucial role in the pathogenesis of ORAS. It has been shown that augmented interferon signaling and inflammasome activation occurred, respectively, in mice expressing OTULIN C129A and in those lacking OTULIN in myeloid cells (Doglio et al., 2023; Heger et al., 2018). Thus, it seems interesting to evaluate those signaling pathways in ORAS patients in addition to TNF signaling in the future, although an anti-IL-1β agent was not effective in ORAS patients in general.

ORAS is triggered by compromised OTULIN function, which is caused by homozygous or compound heterozygotic variants in the *OTULIN* genes (Damgaard et al., 2016, 2019; Nabavi et al., 2019; Zhou et al., 2016; Zinngrebe et al., 2022). In the present study, we show that ORAS can be triggered in an autosomal-dominant fashion by p.R306Q, a dominant-negative OTULIN variant (Fig. 7). A recent study reported that haploinsufficiency of OTULIN is associated with susceptibility to *S*. *aureus* infections (Spaan et al., 2022). It is worth noting that the patient also experienced an *S*. *aureus* infection of the skin (Fig. 1 C and Clinical description), which implies that the characteristic symptom of haploinsufficiency of OTULIN can be observed in the patient, although further analyses are needed to clarify the difference between the two disease statuses. However, some differences in TNF signaling between haploinsufficiency and negative dominance of OTULIN are observed. The level of resistance to TNF-induced cell death shown by fibroblasts from patients with haploinsufficiency is almost the same as that of cells from healthy controls, which may result in an inability to trigger ORAS in haploinsufficient patients (Spaan et al., 2022). Our patient exhibited augmented sensitivity to TNF-mediated cell death, which implies that clinical manifestations resulting from heterozygous OTULIN variants may depend on the level of residual OTULIN function.

## Materials and methods

### Clinical description

The patient is a 9-year-old girl who presented with persistent fever associated with leukocytosis, along with elevated serum CRP levels, sterile pustulosis and erythema nodosum, and impaired physical development.

She was born to non-consanguineous healthy Japanese parents at 33 wk and 1 day of gestation, with a birth weight of 1,436 g. The birth was complicated by an intrauterine infection. Following a vaginal delivery with meconium staining and severe asphyxia, she developed meningitis associated with neutrophil-dominant leukocytosis, as well as elevated serum CRP levels, which responded partially to antibiotic therapy. Although cerebrospinal fluid (CSF) showed polynucleocytosis, blood and CSF cultures did not identify any pathogenic bacteria.

At the age of 1 mo, she developed omphalitis refractory to any antibiotics, although omphalitis cultures revealed methicillin-susceptible *S. aureus*. Therefore, she was referred to our hospital for surgical debridement and further evaluation because the inflammation extended to most of her abdominal wall. Histopathological examination (hematoxylin-eosin [H&E] staining) of a specimen taken during surgical debridement of omphalitis revealed massive infiltration by leukocytes (predominantly neutrophils) and formation of inflammatory granuloma. In addition, TUNEL staining and cleaved caspase-3 staining revealed significant cell death. Immunohistochemistry showed infiltration of TNF-producing cells. Despite surgical debridement and intensive antibiotic therapy with imipenem-cilastatin, doripenem, cefazolin, clindamycin, vancomycin, linezolid, daptomycin, rifampicin, tosufloxacin, clarithromycin, and azithromycin, the systemic and local inflammation persisted. Regardless of these treatments, pustulosis developed at the skin puncture and surgical sites, which was also refractory to antibiotics.

Since the age of 3 mo, the patient suffered three episodes of ARDS associated with pneumonia and thrombocytopenia, which were refractory to antibiotics but responded to high-dose dexamethasone (0.5 mg/kg/day) in combination with sivelestat sodium hydrate. In addition to ARDS, both omphalitis and pustulosis responded to these therapies. However, when the dose of dexamethasone was reduced to 0.1 mg/kg/day, fever relapsed in association with skin erythema, leukocytosis, and elevated CRP levels. Thereafter, several febrile episodes developed in association (or not) with pneumonia, although each episode responded to high-dose prednisolone or methylprednisolone pulse therapy, but relapsed following tapering of prednisolone. The febrile episodes were accompanied by invariably high levels of leukocytosis (up to 1 × 10^8^/μl with neutrophil predominance), thrombocytosis (up to 1 × 10^9^/μl), and CRP levels of 100–500 mg/liter. Nevertheless, no pathogenic bacteria were detected in cultures obtained before treatment with antibiotics. To date, no autoantibodies (such as antinuclear antibodies and rheumatoid factor) have been detected. Severely impaired physical and psychological development was observed during the course of severe inflammation and treatment with high-dose steroids. Serum cytokine analysis revealed significantly elevated levels of IL-1β and IL-6, which resolved following treatment with high-dose dexamethasone or prednisolone. Histopathological examination of the erythema nodosum in her left upper arm at 1 year of age showed diffuse infiltration of the dermis by neutrophils and mononuclear cells producing TNF, with no evident cell death.

At the age of 2 years, the patient was started on etanercept (twice weekly), which completely controlled the inflammation and associated manifestations, including skin erythema. Thereafter, she developed only one febrile episode (at 3 years old) during a human herpes simplex virus 6 infection, which resolved without additional treatment. Following etanercept-induced remission, her physical and psychological development improved. We gradually reduced the dose of prednisolone and she attained steroid-free remission with etanercept therapy.

### Isolation of PBMCs and generation of EBV-transformed cell lines

PBMCs were separated from heparinized peripheral blood samples obtained from a healthy control and the patient by density gradient centrifugation using Histo Paque 1077 (Sigma-Aldrich). EBV-transformed cell lines (EBV-LCLs) were generated by in vitro transformation of human B cells with EBV (Stain B95-8) as described previously (Yamada et al., 2012). EBV-B cells were grown in RPMI-1640 supplemented with 10% fetal bovine serum (FBS) and penicillin/streptomycin. To generate monocyte-derived macrophages (MDMs), CD14-positive cells were sorted using CD14 microbeads (Miltenyi Biotec), suspended in RPMI supplemented with 10% FBS, penicillin/streptomycin, and 5 ng/ml G-CSF, plated (1 × 10^5^ cells per well) into 96-well plates, and incubated for 7 days.

### Cytokine analysis

For cytokine analysis, MDMs and whole blood from seven healthy controls and the patient were exposed to IL-1β for 48 h. Serum and culture supernatants were obtained and stored at −80°C. Cytokine concentrations were measured using an ELISA kit (for IL-6) and LEGENDplex Multiplex assays Inflammation panel 1 (BioLegend), as described previously (Ueki et al., 2023). Fluorescent beads were detected by a CytoFLEX apparatus (Beckman Coulter). Data represent the mean of triplicate wells.

### Histology, immunohistochemistry, and TUNEL assay

Surgically resected omphalitis tissue, as well as sections of inflamed skin, were fixed with formalin and embedded in paraffin. Immunochemistry was performed using antibodies specific to caspase-3. Immunohistochemistry and the TUNEL assay were performed as previously described (Cho et al., 2018).

### Sample preparation, cloning, and direct sequence analysis

Genomic DNA was obtained from whole blood using Sepagene (Sekisui Chemical Co). RNA was obtained from peripheral mononuclear cells as previously described (Yamada et al., 2012). cDNA was generated using a PrimeScript RT reagent kit (Takara). Full-length *OTULIN* cDNA was amplified using primers F 5′-AAG​TTC​TGT​TTC​AGG​GCC​CGA​TGA​GTC​GGG​GGA​CTA​TGC​CCC-3′; R 5′-ATG​GTC​TAG​AAA​GCT​TTA​TAG​ACT​GGT​CTC​CTC​ACA​CAC​TCT​G-3′. Cloning of amplified cDNA was performed using a TOPO TA cloning kit (Thermo Fisher Scientific). These kits were used in accordance with the manufacturer’s instructions. The coding exon and exon–intron boundary were amplified using the primers listed in Table S6. Sequence analysis was performed at FASMAC Co. Ltd. (Atsugi) using an Applied Biosystems 3730xl DNA Analyzer.

### Sample preparation and WES

Genomic DNA was isolated from peripheral blood leukocytes using QuickGene 610L (Wako). It was then captured using the SureSelect Human All Exon v5 (50 Mb) or v6 (60 Mb) Kit (Agilent Technologies) and sequenced on an Illumina HiSeq2500 (Illumina) with 101-bp paired-end reads. Exome data processing, variant calling, and variant annotation were performed as previously described (Seyama et al., 2023). The average read depth of protein-coding regions was >60× and at least 90% of target bases were sequenced by 10 or more reads. Common single-nucleotide polymorphisms with minor allele frequencies ≥1% in dbSNP 137 and variants observed in more than 5 of 575 in-house ethnically matched control exomes were filtered out. Among the remaining rare variants, focus was placed on amino acid-altering or splicing-affecting variants. Candidate variants were confirmed by Sanger sequencing of PCR products using an ABI PRISM 3500xl genetic analyzer (Life Technologies) using genomic DNA from the patient and parents as a template.

### Plasmids, reagents, and antibodies

The open reading frames of human OTULIN were amplified by RT-PCR. Two-step PCR was then used to generate OTULIN mutants. Briefly, cDNAs were ligated to the appropriate epitope tag sequences and then cloned into pcDNA3.1 (Thermo Fisher Scientific), pGEX-6p1 (Cytiva), pMAL-c2x (New England Biolabs), pMXs-IRES-blasticidin, or pEU-E01 His-TEV (CellFree Science) vectors. pSpCas9(BB)-2A-Puro (pX459) (#48139; Addgene) and pEF1-ABEmax-P2A-GFP (#112101; Addgene) were used for KO and base editing of OTULIN, respectively. To generate pEF1-ABEmax-P2A-GFP, the promoter region of pCMV-ABEmax-P2A-GFP was replaced with the EF1 promoter. All antibodies used are listed in Table S5.

### Cell culture, transfection, retroviral expression, and generation of OTULIN KO cells

HeLa-mCat, HEK293, and HEK293T cells were grown in DMEM supplemented with 10% FBS and penicillin/streptomycin. Cells were transfected with plasmids using Lipofectamine 2000 (Thermo Fisher Scientific) or PEI-max (Polysciences). To knock out OTULIN, HeLa and HEK293 cells were transfected with pX459, which encodes a guide RNA sequence targeting OTULIN (5′-GAT​CAC​CAC​GGA​CTC​GCC​GTA-3′). After transfection with Lipofectamine 2000, cells were selected by incubation with puromycin for 2 days. Isolated colonies were verified as OTULIN KO cells by immunoblotting with anti-OTULIN antibody. For retroviral infection, pMXs-IRES-puro- or pMXs-IRES-blasticidin-encoding OTULIN mutants was transfected into platE or GP2-293 packaging cells, as previously described (Fuseya et al., 2020). The resultant viruses were used to infect OTULIN KO HeLa or HEK293 cells, and stably transduced cells were selected using puromycin or blasticidin.

### Immunoblot analysis

Cells were lysed in lysis buffer containing 50 mM Tris-HCl (pH 8.0), 150 mM NaCl, 1% Triton X-100, 2 mM PMSF, 2 mM EDTA, 2 mM imidazole, and 10 mM NaF, with or without 5 mM N-ethylmaleimide and 7.5 mM cysteine. Lysates were clarified by centrifugation at 20,000 × *g* for 20 min at 4°C. The proteins in the lysates were separated by SDS-PAGE and transferred to polyvinylidene difluoride (PVDF) membranes. After blocking in Tris-buffered saline containing 0.1% Tween-20 and 5% (wt/vol) nonfat dry milk, the membrane was incubated with the appropriate primary antibodies, followed by appropriate secondary antibodies (see Table S5). The membranes were visualized using enhanced chemiluminescence and a LAS4000mini instrument (GE Healthcare).

### Purification of recombinant OTULIN and DUB analysis

The GST-OTULIN mutants and MBP-OTULIN mutants were expressed in *E. coli* BL21-CodonPlus(DE3) cells (#230280; Agilent). GST-fusion proteins and MBP-fusion proteins were purified using glutathione Sepharose (#17513202; Cytive) and amylose resin (New England BioLabs), respectively. GST-OTULIN mutants were cleaved by overnight incubation at 4°C with PreScission Protease. The supernatants were desalted on a PD-10 column using DUB assay buffer (20 mM Tris-Cl [pH 7.5] and 5 mM DTT). MBP-OTULIN mutants were eluted by maltose (10 mM)-containing buffer. The elution products were desalted on a PD-10 column using SPR analysis buffer (20 mM HEPES [pH 7.5], 150 mM NaCl). GST-diUb and His-diUb were prepared and purified as described previously (Kirisako et al., 2006). For the time-dependent DUB assay, 15 μl of the DUB reaction mixture containing 700 ng of His-diUb (final concentration, 2.5 µM), 15 ng of OTULIN mutant (final concentration, 25 nM), 20 mM Tris-Cl (pH 7.5), and 5 mM DTT were warmed at 37°C for the indicated times. The reaction was stopped by adding 5 μl of 4× SDS sample buffer. Samples were boiled at 95°C for 5 min and separated by SDS-PAGE in MES running buffer, followed by staining with Coomassie Brilliant Blue (CBB). For the OTULIN concentration-dependent DUB assay, 15 μl of DUB reaction mixture containing GST-diUb (final concentration, 2.5 µM), OTULIN mutants (final concentration, 10 or 300 nM), 20 mM Tris-Cl (pH 7.5), and 5 mM DTT were warmed at 37°C for 3 min. The reaction was stopped by adding 5 μl of 4× SDS sample buffer. Samples were then boiled at 95°C for 5 min, separated by SDS-PAGE, transferred to a PVDF membrane, and detected by an anti-GST antibody.

### 3D structural modeling

The structures of OTULIN were obtained from PDB IDs 3ZNV (free form) and 5OE7 (diUb-bound form). AlphaFold (Jumper et al., 2021) was used via the Google Colab notebook. Visualization and analysis were carried out in PyMOL software version 2.5.1 (Schrödinger).

### SPR analysis

The binding affinity between GST-diUb and the MBP-OTULIN mutants was measured on a Biacore 3000 (Cytiva). Briefly, GST-diUb in 10 mM HEPES (pH 7.4) buffer containing 150 mM NaCl and 0.05% (vol/vol) surfactant P20 at 25°C was immobilized on a CM5 sensor chip using an anti-GST antibody and the GST capture kit (Cytiva).

### Immunoprecipitation and DUB analysis

Cells were lysed with lysis buffer containing 10 mM Tris-HCl (pH 7.5), 150 mM NaCl, 0.2% NP-40, 10% glycerol, 2 mM PMSF, 2 mM EDTA, 2 mM imidazole, 10 mM NaF, 5 mM *N*-ethylmaleimide, and 7.5 mM cysteine, and centrifuged at 20,000 × *g* for 20 min at 4°C.

Immunoprecipitation of myc-OTULIN was performed with an anti-myc antibody and Dynabeads protein L (#88849; Thermo Fisher Scientific). Immunoprecipitation of endogenous LUBAC was performed with an anti-Sharpin antibody and Dynabeads protein A (#10002D; Thermo Fisher Scientific). TNFR Complex I was immunoprecipitated from cell lysates by a FLAG-His-TNF-α antibody (100 ng/ml) (Fuseya et al., 2020), an anti-FLAG M2 antibody, and Dynabeads protein A. Briefly, magnetic beads were incubated with cell lysates overnight at 4°C, followed by three washes with lysis buffer and TBS. For DUB analysis, OTULIN OTU domain (1 μg) in 20 μl buffer containing 20 mM Tris-HCl (pH 7.5) and 5 mM dithiothreitol (DTT) was added to the immunoprecipitated sample and incubated at 37°C for 30 min. Immunoprecipitants were analyzed by immunoblotting.

### Immunoprecipitation of TNFR Complex II

Cells were pretreated with 10 μM Z-VAD-FMK (Peptide Institute) for 1 h and then stimulated with 10 ng/ml TNF-α (Wako Chemicals) and 20 μg/ml CHX (Calbiochem) for the indicated times. Cells were then lysed with lysis buffer (30 mM Tris-HCl [pH 7.5], 120 mM NaCl, 1% Triton X-100, 10% glycerol, 2 mM PMSF, 2 mM EDTA, 2 mM imidazole, and 10 mM NaF), followed by centrifugation at 20,000 × *g* for 20 min at 4°C. The cleared lysates were immunoprecipitated with an anti-caspase-8 antibody and Dynabeads protein A. Immunoprecipitants were analyzed by immunoblotting.

### Generation of iPSCs from the patient

The generation of iPSCs from the patient was performed as described previously (Nakagawa et al., 2014). Control iPSC lines were provided by the RIKEN BioResearch Center through the National Bio-Resource Project of the Ministry of Education, Culture, Sports, Science, and Technology, Japan.

Human pluripotent stem cells were differentiated into ectoderm, mesoderm, and endoderm lineages using the STEMdiff Trilineage Differentiation Kit (STEMCELL Technologies). After reaching 70–80% confluence, the cells were harvested by the addition of TrypLE Select Enzyme (Thermo Fisher Scientific). A single cell suspension was prepared in mTeSR1 medium (STEMCELL Technologies) containing 10 mmol/liter Y-27632 (Wako Chemicals) and plated at 3.0 × 10^5^ cells/well on 6-well plates coated with Matrigel (BD Biosciences) for differentiation into ectoderm or mesoderm, and at 4.0 × 10^5^ cells/well for differentiation into endoderm. Cells were harvested on day 7 (ectoderm) or day 5 (mesoderm and endoderm). Undifferentiated pluripotent stem cells and human pluripotent stem cell-derived ectoderm, mesoderm, and endoderm (1.0 × 10^6^ cells each) were fixed for 20 min at 4°C with 4% paraformaldehyde in PBS (4% paraformaldehyde/PBS) and then washed twice with staining medium containing PBS/2% FBS. Samples were permeabilized for 15 min at room temperature with BD Perm/Wash buffer (BD Biosciences) and then stained for 1 h with the fluorescently conjugated primary antibodies (see Table S5). Samples were washed with BD Perm/Wash buffer twice and suspended in a staining medium. Finally, cells were acquired by an LSR flow cytometer (BD Biosciences). Data were analyzed and graphs were generated by FlowJo software (FlowJo).

### Measurement of LDH release

LDH release was measured using a Cytotox96 Non-Radioactive Cytotoxicity Assay kit (Promega). Briefly, cells were seeded on 96-well plates at 2 × 10^4^ cells per well and then treated with TNF (10 ng/ml) and CHX (20 μg/ml). After culture for the indicated periods, the media were collected. LDH levels in culture media were determined by measuring the absorbance at 490 nm on a SpectraMax M5 (Molecular Devices).

### Annexin-V and TO-PRO-3 stain

Briefly, cells were seeded on six-well plates at 1 × 10^5^ cells per well and then treated with TNF (10 ng/ml) and CHX (20 μg/ml). After culture for the indicated periods, the cells were collected and washed with Annexin V–binding buffer (BD Pharmagen) twice. Cells were resuspended with Annexin V–binding buffer and then stained with Annexin-V (BioLegend) and TO-PRO-3 (Thermo Fisher). The percentage of Annexin-V or TO-PRO-3–positive cells was measured on a FACS Canto II.

### Culture media and reagents of iPS cells

The induction of hNCCs (neural crest cells) from iPSCs and MSCs from hNCCs were performed as previously described (Fukuta et al., 2014). Briefly, iPSCs were cultured with SB431542 (10 μM) and CHIR99021 (10 μM) in CDM, which contains Iscove’s modified Dulbecco’s medium/Ham’s F-12 1:1, 1× chemically defined lipid concentrate (GIBCO), 15 µg/ml apo-transferrin (Sigma-Aldrich), 450 µM monothioglycerol (Sigma-Aldrich), 5 mg/ml purified BSA (99% purified by crystallization; Sigma-Aldrich), 7 µg/ml Insulin (Wako Chemicals), and penicillin/streptomycin. Culture dishes were coated with growth factor–reduced Matrigel (BD Biosciences) or fibronectin (Millipore).

### Fluorescence-activated cell sorting (FACS)

Cells were harvested at 7 days after induction and sorted according to the expression of p75 using a FACSAriaII cytometer (BD Biosciences). Two peaks, p75^low^ and p75^high^, were detected and the efficiency of hNCC induction was evaluated based on the fraction of p75^high^ cells. EGF (R&D) and FGF2 were used to maintain hNCCs in culture.

### Base editing of iPSCs

To change OTULIN L152 to Pro, or Q306 to Arg, iPSCs were electroporated with pEF1-ABEmax-P2A-GFP (encoding a guide RNA sequence targeting L152 to Pro or Q306 to Arg by Nepa21) in an electroporator (NepaGENE). Cells were then selected with GFP. Isolated colonies were verified by genome sequencing. The primers used are listed in Table S6.

### Induction of MSCs from hNCCs

The cell culture medium was replaced with αMEM (Nacalai Tesque) supplemented with 10% FBS. Cell morphology began to change at ∼4 days after induction. Cells were passaged every week using 0.25% trypsin-EDTA (GIBCO) and a reseeding density of 1 × 10^4^ cells/cm^2^.

### Heavy-labeled recombinant OTULIN mutants

Wheat germ cell-free expression kits (CellFree Sciences) were used to generate isotope-labeled OTULIN mutant proteins. In vitro translation was carried out using WEPRO 9240 wheat germ extract. The synthesized isotope-labeled OTULIN mutant proteins were mixed with 60 μl of 20% Ni-NTA (#1018240; QIAGEN) and 20 mM imidazole. After incubation for 90 min at 4°C, the beads were washed four times with 600 μl of wash buffer containing 20 mM sodium phosphate (pH 8.0), 300 mM NaCl, 50 mM imidazole, and 0.05% Triton X-100. The beads were then eluted twice with 40 μl of an elution buffer containing 20 mM sodium phosphate (pH 8.0), 300 mM NaCl, and 500 mM imidazole. Finally, the elution products were quantified by SDS-PAGE and CBB staining with BSA as a standard.

### Mass spectrometry analysis of OTULIN mutants

Cell lysates (100 μg) from MSCs were mixed with 0.5 pmol of each heavy-labeled recombinant OTULIN mutant. Analytes were separated by SDS-PAGE and stained with Bio-Safe Coomassie (Bio-Rad). Gel regions corresponding to 35–63 kDa were excised and sliced. The gel pieces were washed in 50 mM AMBC/30% acetonitrile (ACN) followed by 50 mM AMBC/50% ACN and then dehydrated in 100% ACN. Proteins were digested for 16 h at 37°C with 20 ng/μl sequence-grade trypsin (Promega) in 50 mM AMBC/5% ACN, (pH 8.0). The digested peptides were extracted three times with 0.1% trifluoroacetic acid (TFA)/70% and concentrated using a vacuum centrifuge. The proteins were then resuspended in 0.1% TFA/0.05% H_2_O_2_ and incubated overnight at 4°C to oxidize methionine. For liquid chromatography-tandem mass spectrometry, an Easy nLC 1200 (Thermo Fisher Scientific) was connected inline to an Orbitrap Fusion LUMOS (Thermo Fisher Scientific), and the peptides were separated on a C18 analytical column (IonOpticks, Aurora Series Emitter Column: AUR2-25075C18A, 25 cm × 75 μm; 1.6 μm FSC C18 with a nanoZero fitting) using an 85 min gradient (solvent A, 0.1% formic acid [FA]; solvent B, 80% ACN/0.1% FA). An Orbitrap Fusion LUMOS instrument was operated in targeted MS/MS mode using Xcalibur software, and the peptides were fragmented using higher-energy collisional dissociation with a normalized collision energy of 28. Fragments were detected by an Orbitrap. Data were processed using the PinPoint software (Thermo Fisher Scientific), and peptide abundance was calculated from the integrated area under the curve for the selected fragment ions. The mutant-specific parental and fragmented ions are summarized in Table S4.

### Purification of linear ubiquitin by M1-Tandem Ubiquitin Binding Entity (TUBE)

Halo-tagged linear ubiquitin chain specific (M1-TUBE) was purified as described previously (Jo et al., 2020). To measure the amount of linear ubiquitin in MSCs, 100 µg of cell lysate was incubated for 4 h at 4°C with 2 µg of M1-TUBE and 5 μl of equilibrated MagneHalo Tag beads (Promega) in 50 mM Tris-HCl (pH 8.0), 150 mM NaCl, 0.05% Triton X-100. The precipitates were washed three times with the same buffer, boiled at 95°C for 5 min, and analyzed by immunoblotting.

### Luciferase activity

To measure NF-κB activation, HEK293T cells were co-transfected with pGL4.32 (Luc2p/NF-κB-RE/Hygro) and pGL4.74 (hRLuc/TK) (Promega), together with various expression plasmids. At 24 h after transfection, cells were lysed, and luciferase activity was measured by a Lumat Luminometer (Berthold) using the Dual-Luciferase reporter assay system (Promega).

### Quantitative RT-PCR (qPCR) analysis

Total RNA was isolated using the RNeasy Micro or Mini Kit (Qiagen). DNase-treated RNA (20–200 ng) was reverse-transcribed using the high-capacity RNA-to-cDNA Kit (Applied Biosystems). Real-time PCR was performed using Power SYBR Green PCR master mix (Applied Biosystems) and an ABI 7900 Real-time PCR system (Applied Biosystems). All gene expression levels were normalized against the corresponding levels of β-actin mRNA. The primers used for qPCR are listed in Table S6.

### Statistics

Data are presented as the mean ± SD or SEM. Microsoft Excel and Prism were used to generate the graphs and for statistical analyses. Data were analyzed using a one-way analysis of variance. The statistical methods used are described in each of the figure legends. Differences are indicated as ns (not significant; P > 0.05), *P < 0.05, **P < 0.01, or ***P < 0.001, unless indicated otherwise in the figures.

### Study approval

The study was approved by the committee on ethics of Hokkaido University Faculty of Medicine and Graduate School of Medicine (14-002 for the genetic and molecular analysis, and 17-028 for the establishment and analysis of iPSCs). Written informed consent was obtained from the guardians of the patient and the healthy volunteers before the study.

### Online supplemental material

Fig. S1 shows the structural modeling of the OTULIN R306Q substitution. Fig. S2 includes additional analyses of OTULIN KO HeLa cells expressing indicated OTULIN variants. Fig. S3 shows the characterization and analyses of patient-derived iPSCs. Fig. S4 includes additional analyses of patient iPSC–derived MSCs. Fig. S5 shows the dominant-negative effect of the OTULIN R306Q variant and a schematic model of the dominant-negative function of the p.R306Q variant. Table S1 shows analyses of cytokines in serum from the patient. Table S2 shows genetic variant lists identified by WES. Table S3 shows the comparison of reported ORAS cases. Table S4 shows a list of mutant-specific parental and fragmented ions used for MS/MS analysis in Fig. 5 B. Table S5 shows a list of antibodies used in this study. Table S6 shows a list of PCR primers used in this study.

## Supplementary Material

Table S1 shows analysis of cytokines in serum from the patient.

Table S2 shows genetic variants identified by WES.

Table S3 shows comparison of ORAS cases.

Table S4 lists mutant-specific parental and fragmented ions.

Table S5 lists antibodies used in this study.

Table S6 lists PCR primers used in this study.

SourceData F2 contains original blots for Fig. 2.

SourceData F3 contains original blots for Fig. 3.

SourceData F4 contains original blots for Fig. 4.

SourceData F5 contains original blots for Fig. 5.

SourceData F6 contains original blots for Fig. 6.

SourceData F7 contains original blots for Fig. 7.

SourceData FS2 contains original blots for Fig. S2.

SourceData FS3 contains original blots for Fig. S3.

SourceData FS4 contains original blots for Fig. S4.

SourceData FS5 contains original blots for Fig. S5.

## Data Availability

The data underlying figures are available in the published article and its online supplemental material.
